# Pasture Names with Romance and Slavic Roots Facilitate Dissection of Y Chromosome Variation in an Exclusively German-Speaking Alpine Region

**DOI:** 10.1371/journal.pone.0041885

**Published:** 2012-07-27

**Authors:** Harald Niederstätter, Gerhard Rampl, Daniel Erhart, Florian Pitterl, Herbert Oberacher, Franz Neuhuber, Isolde Hausner, Christoph Gassner, Harald Schennach, Burkhard Berger, Walther Parson

**Affiliations:** 1 Institute of Legal Medicine, Innsbruck Medical University, Innsbruck, Austria; 2 Institute of Lexicography of Austrian Dialects and Names, Austrian Academy of Sciences, Vienna, Austria; 3 Institute of Legal Medicine, University of Salzburg, Salzburg, Austria; 4 Central Institute for Blood Transfusion & Immunological Department, Innsbruck, Austria; 5 Blood Transfusion Service Zürich, SRC, Schlieren, Switzerland; Erasmus University Medical Center, Netherlands

## Abstract

The small alpine district of East Tyrol (Austria) has an exceptional demographic history. It was contemporaneously inhabited by members of the Romance, the Slavic and the Germanic language groups for centuries. Since the Late Middle Ages, however, the population of the principally agrarian-oriented area is solely Germanic speaking. Historic facts about East Tyrol's colonization are rare, but spatial density-distribution analysis based on the etymology of place-names has facilitated accurate spatial mapping of the various language groups' former settlement regions. To test for present-day Y chromosome population substructure, molecular genetic data were compared to the information attained by the linguistic analysis of pasture names. The linguistic data were used for subdividing East Tyrol into two regions of former Romance (A) and Slavic (B) settlement. Samples from 270 East Tyrolean men were genotyped for 17 Y-chromosomal microsatellites (Y-STRs) and 27 single nucleotide polymorphisms (Y-SNPs). Analysis of the probands' surnames revealed no evidence for spatial genetic structuring. Also, spatial autocorrelation analysis did not indicate significant correlation between genetic (Y-STR haplotypes) and geographic distance. Haplogroup R-M17 chromosomes, however, were absent in region A, but constituted one of the most frequent haplogroups in region B. The R-M343 (R1b) clade showed a marked and complementary frequency distribution pattern in these two regions. To further test East Tyrol's modern Y-chromosomal landscape for geographic patterning attributable to the early history of settlement in this alpine area, principal coordinates analysis was performed. The Y-STR haplotypes from region A clearly clustered with those of Romance reference populations and the samples from region B matched best with Germanic speaking reference populations. The combined use of onomastic and molecular genetic data revealed and mapped the marked structuring of the distribution of Y chromosomes in an alpine region that has been culturally homogeneous for centuries.

## Introduction

The small Tyrolean district of Lienz has been commonly referred to as “East Tyrol” since the end of World War I. Due to East Tyrol's high-alpine topology, its population (∼50,000) is largely restricted to the mountain-valleys. A mere 8% (165.5 km^2^) of the district's total area (2,020 km^2^, http://tirolatlas.uibk.ac.at) are permanently settled. The principal occupations are in agriculture (mostly dairy farming, stock breeding, and forestry) and the total arable land amounts to 934.8 km^2^ (46%). Notably, in 1986, pastures (“Almen”) accounted for 44% of the total Tyrolean area (http://www.statistik.at), a number that clearly indicates their significance in agriculture. By definition, pastures comprise agricultural areas (and objects) that are, due to the climatic conditions arising from their high altitude, subject to a reduced vegetation period in which the keeping of grazing animals is suitable. Consequently, farming at pastures occurs only in a seasonal fashion, mostly during the summer months. In the Alpine region transhumance played a vital role since the early times of settlement.

The beginning of industrialization did not leave significant marks in East Tyrol. Although small craft enterprises developed in the city of Lienz and other rural areas, they never reached the importance of agriculture. It was only in the second half of the 20^th^ century that industrial manufacturing of e.g. cooling devices and electrical appliances were found in the region around East Tyrol's capital Lienz and Heinfels, respectively. And finally, the tourism and service sectors gained economical significance in the late 19^th^ century as a result of improved transportation connections.

With regard to the landscape's paramount role in human migration, settlement and occupation, East Tyrol can be considered a typical alpine region. However, unlike other European alpine regions, East Tyrol is unique in that members of the Romance, the Slavic and the Germanic speaking language groups simultaneously occupied this area for several centuries.

To gain a more complete understanding of the complex cultural and demographic processes that shaped the present-day East Tyrolean population, an examination of the sparse historical, archaeological, and linguistic facts is necessary.

At about 15 before Common Era the “Kingdom of Noricum”, to which the area of East Tyrol belonged, accepted the suzerainty of the Roman Empire [1]. The most important city in the study area was Aguntum, a pre-Roman settlement that received its town charter “Municipium Claudium Aguntum” at about 41/54 [2]. The time of first Roman settlement falls within this period as well. The first reference to Slavic and Germanic (Bavarian) presence in the area of East Tyrol is given by the Lombardic historian Paulus Diaconus, who reports a battle between these two ethnic groups in 610 [3]. The fighting took place near the city of Aguntum, which was completely destroyed and never rebuilt (its ruins are located ∼4 km to the east of the town of Lienz). The battle was particularly important as it stopped the westbound Slavic expansion [1] and, as a result, the southwestern part of East Tyrol was never populated by the Slavic language group. Romance speaking inhabitants, however, remained in the more rural regions of East Tyrol.

The situation from the middle of the 7^th^ century onward was characterized by a more or less peaceful coexistence of the three ethnic groups with steadily growing colonization pressure from the Bavarian people [4].

**Figure 1:**
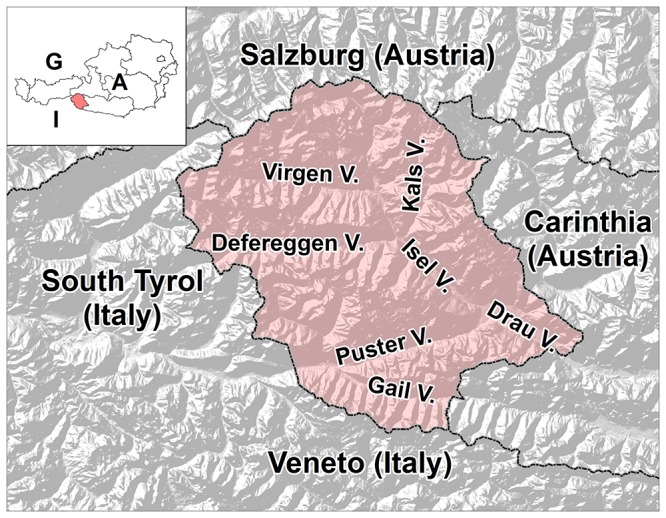
Map of East Tyrol. The Austrian political district of Lienz (“East Tyrol”) is highlighted in orange. **A**: Austria, **G**: Germany, **I**: Italy.

The point in time at which the Romance and Slavic languages were completely replaced by the Bavarian dialect of the Germanic language group can likely only be established with linguistic methods. Conversion of older field names into German and linguistic borrowings (foreign words) reveals that at the end of the 14^th^ century Romance speaking people were restricted to very few isolated areas (especially in Kals and in the very south of East Tyrol at the Venetian border). The last traces of the Slavic speaking population can be found at about the same time in the north of East Tyrol (especially in the Virgen and Kals valleys, Figure 1). Since then, the entire East Tyrolean population has spoken only German, or more specifically, a Bavarian dialect. Neither a Slavic (Slovenian) minority language group like in the adjacent province of Carinthia (Austria) nor a Romance (Ladin) minority language group like in the adjacent province South Tyrol (Alto Adige, Italy) is to be found (Fig. 1).

This transition to a linguistically homogeneous population could be explained by either cultural diffusion or by the range expansion of the Germanic speaking people and replacement of the long-term resident Slavic and Romance speaking populations over time. In the case of cultural diffusion, genetic signatures of the originally non-Germanic speaking people might be still detectable in East Tyrol.

The analysis of human Y chromosome variation is a well established tool for addressing questions regarding human history on the basis of present-day genetic diversity. The non-recombining part of the human Y chromosome (NRY) is constitutively haploid and, barring mutations, transmitted clonally along stable paternal lineages. Due to its smaller effective population size as compared to autosomes, the NRY shows a higher susceptibility to genetic drift, which in turn leads to population differentiation. Furthermore, patrilocality is common in modern human societies. This social phenomenon results in increased geographical clustering of Y chromosomes and enhanced local differentiation, even at the micro-geographical scale.

Here, we attempt to shed light on the significance of complex historical events on the present-day Y chromosome variation in the highly structured alpine area of East Tyrol by linking molecular genetic data with a linguistic perspective.

## Materials and Methods

### Biological Samples

The study was reviewed and approved by the ethics commission of the Innsbruck Medical University (study classification number UN2598, session number 241/4.5). Voluntary blood donors got detailed information about the study in advance as well as on-site and provided both written consent and personal plus genealogical data (full names, birth dates and the family's places of residence at the time of birth of the donor, his father and paternal grandfather) prior to participation. The personal data as well as all associated genotyping results were stored and managed by means of a dedicated database developed and held in-house. Blood samples (∼5 ml) from healthy West Eurasian men living in the Tyrolean political district Lienz, which equates to East Tyrol, were obtained in the course of a single blood collection campaign in the city of Lienz that was organized by the “Central Institute for Blood Transfusion and Immunological Department Innsbruck” in the year 2007.

### Onomastics

Several name concepts were used throughout the text. The term “place name” was used in a broad sense and, hence, included all names that applied to any topographic feature. As opposed to this, the term “field name” was used for names denominating unpopulated areas. Finally, “surname” refers to that part of a person's name, which is passed on in a patrilineal way. It was also used as a synonym to “family name”.

The surnames of the men participating in this study and 853 East Tyrolean pasture names that were collected and analyzed during the course of a separate project, constituted the onomastic basis of this study. To arrive at the etymon (word root) of a particular name, and therefore to assign it to a certain language group, a diachronic linguistic analysis was performed on the historical forms in written sources and the pronunciation of the names in the dialect. These results were verified by comparing them to reality according to [4]. Though not every etymon could be determined to its lexical units and former semantic meaning, the language group could be identified in most cases by formal criteria that uses, for example, the pre- and suffices derivation mechanisms, and other linguistic features [5].

### Pasture Name Density Maps

A standard point density procedure implemented in the ArcMap GIS-program (version 9.3.1, ESRI, Kranzberg, Germany) was used for the calculation of the density maps comprising a grid. For each grid cell the magnitude/unit-area of pasture names falling within its circular neighborhood of 10 kilometers was calculated [6].

### DNA Extraction and Storage

Total genomic DNA (gDNA) was extracted in the 96-well format from 200 µl blood-aliquots using the nexttec genomic DNA isolation kit for blood (nexttec Biotechnologie, Leverkusen, Germany) according to the manufacturer's instructions. The DNA samples were stored in racked Matrix 2D barcoded polypropylene storage tubes (Thermo Fisher Scientific, Hudson, NH, USA) at −80°C until further use. For automated liquid handling in Y-chromosomal genotyping a Tecan Genesis robot was used (Tecan Group, Männedorf, CH).

### Primer Design

Oligonucleotides for polymerase chain reaction (PCR) amplification and single nucleotide primer extension (SNPE) used for the genotyping of 27 phylogenetically informative single nucleotide polymorphisms (SNPs) on the NRY (SNPE approach: M9, M17, M45, M78, M89, M96, M170, M173, M201, M223, M253, M269, M304, M343, P15, P37, SRY_10831_, U106/S21, and U152/S28; Sanger-type sequencing: L11/S127, L23/S141, M20, M70, M242, M412/S167, M529/S145, and S116) were either taken from the literature or newly designed using the freeware programs Primer-BLAST [7] and Primer3Plus [8]. For a full list of synonymous Y-SNP designations refer to Table S1. All primer sequences can be found in Table S2 and Table S3. For all primers the PCR/SNPE multiplexing compatibility was tested with the AutoDimer software [9]. For marker P37, primers were designed that prevented co-amplification of its X-chromosomal gametologue [10], which mimics the derived Y-chromosomal SNP allele C. By means of sequencing analysis, the actual position of the Y-SNP P15 was found to be 2 base pairs (bp) upstream from that reported in [11], [12]. The 3′end of the P15 SNPE primer was placed accordingly. The sequences of all PCR amplicons are given in Table S4.

All primers were purified by the manufacturer (Microsynth, Balgach, Switzerland) and their concentrations were verified in-house by UV absorbance reading at λ = 260 nm.

The phylogenetic tree defined by the Y-SNP panel used in this study is shown in Fig. S1. For the sake of readability and ease of comparability, haplogroups were designated by their respective single letter major haplogroup name followed by the most derived biallelic marker. Asterisks indicate that for a particular haplogroup all analysed subgroup defining Y-SNPs exhibited their ancestral base state.

### PCR-Multiplex Amplification for SNPE

To determine the optimum concentrations of the PCR primers used for the co-amplification of the 19 SNPE target sequences, ion pair reversed-phase high-performance liquid chromatography coupled with electrospray ionization time-of-flight mass spectrometry [13]–[15] was used for the direct analysis of the PCR-multiplex products in initial experiments. The 10 µl amplification reactions comprised of 1× KOD buffer (Novagen, Gibbstown, NJ, USA), 2.5 µg non-acetylated bovine serum albumin, 5% (w/v) trehalose (both Sigma-Aldrich, St. Louis, MO, USA), 1 mM MgSO_4_, 200 µM of each deoxynucleoside triphosphate, 0.2 units KOD hot start DNA polymerase (all Novagen), and 2 µl undiluted gDNA extract. The assay concentrations of the PCR primers are given in Table S2.

19-plex PCR, generating amplicon sizes between 81 and 225 bp (Table S4), was carried out in 96-well polypropylene PCR plates in conventional thermal cyclers using an initial heat soak at 94°C for 2 min, followed by 35 cycles of 95°C for 15 s, 58°C for 1 min, and 72°C for 1 min. The final extension step at 72°C was extended by 10 min.

### Single Nucleotide Primer-Extension Multiplex

Prior to SNPE, unincorporated primers and deoxynucleoside triphosphates were enzymatically degraded by adding 1 µl ExoSAP-IT (USB, Cleveland, OH, USA) to 2.5 µl of multiplex PCR products and incubating these reactions for 90 min at 37°C and subsequent enzyme inactivation at 80°C for 20 min. Finally, 9 µl of H_2_O were added. The SNPE reactions were conducted in a total volume of 10 µl containing 2.5 µl 5× sequencing buffer, 2.5 µl SNaPshot multiplex kit (both Applied Biosystems, Foster City, CA, USA), 400 µM spermidine, 200 µM spermine (both Sigma-Aldrich), 5% (w/v) trehalose, 15 nM each SNPE primer (Table S3), and 1 µl of the enzymatically treated and 5-fold diluted PCR products. The SNPE primers were dissolved in 1 mM Tris (pH 8.0 at room temperature), 160 mM ammonium sulphate (Sigma-Aldrich) [16] and premixed. The assay-concentration of (NH_4_)_2_SO_4_ amounted to 12 mM. The thermal cycling protocol consisted of 30 cycles of 96°C for 10 s, 50°C for 5 s, and 60°C for 30 s. SNPE was followed by de-phosphorylation of unincorporated dideoxynucleoside triphosphates for 1 hour at 37°C using 1 unit of shrimp alkaline phosphatase (USB) per 10 µl reaction, and subsequent inactivation of the enzyme at 80°C for 20 min.

### Electrophoretic Separation and Data Analysis

Two microliters of enzymatically treated SNPE product were transferred into 20 µl deionized formamide containing 0.5 µl GeneScan 120 LIZ internal lane size standard (Applied Biosystems) and heat denatured at 95°C for 3 min. Laser induced fluorescence capillary electrophoresis was performed on an ABI 3100 Genetic Analyzer, using a 36 cm capillary array, POP-6 (all Applied Biosystems) as sieving matrix, and default conditions for sample loading and separation. Raw-data were analyzed with the GeneMarker HID v1.70 computer program (SoftGenetics, State College, PA, USA). Automated Y-SNP haplogroup assignments were performed by means of the database used for data storage and management. Haplogroup frequencies below 5% were considered non-informative.

### Sanger Sequencing

As an add-on to 19-plex SNPE typing we further analyzed both all Y chromosomes featuring M9 as the most derived Y-SNP and all haplogroup R-M269 derived Y chromosomes with ancestral base states at the U106/S21 and U152/S28 SNP loci. To type the biallelic markers L11/S127, L23/S141, M20, M70, M242, M412/S167, M529/S145, and S116 (Fig. S1, Table S1), direct Sanger-type sequencing analysis of PCR products was performed. Singleplex reaction mixes consisted of 1× PCR buffer II (Applied Biosystems), 2.5 µg non-acetylated bovine serum albumin (Sigma-Aldrich), 1.5 mM MgCl_2_, 200 µM of each deoxynucleoside triphosphate, 2 units AmpliTaq Gold DNA polymerase (all Applied Biosystems), and 2 µl undiluted gDNA extract. The assay concentrations of the primers used for amplification are given in Table S2.

Singleplex PCRs, generating amplicon sizes between 305 and 402 bp (Table S4), were carried out in 96-well polypropylene PCR plates in conventional thermal cyclers using for all target sequences an initial denaturation at 95°C for 10 min, followed by 40 cycles of denaturation at 95°C for 15 s, primer-annealing at 64°C for 45 s, and primer extension at 72°C for 1 min. For marker L11/S127, a two-step thermal cycler protocol was used with annealing/extension at 72°C for 90 s. The final extension step at 72°C was extended by 10 min in all amplifications.

Post-PCR treatment and cycle sequencing were performed following the experimental procedures detailed in [17]. All sequencing primers (Table S2) were used at an assay-concentration of 160 nM.

### Y-Chromosomal Short Tandem Repeat Haplotyping

Seventeen locus Y-chromosomal short tandem repeat (Y-STR) haplotypes were determined from all samples in this study with the AmpFlSTR Yfiler PCR amplification kit (Applied Biosystems) as described in [18]. To test for potential (pseudo) null alleles in samples yielding only a single peak for the duplicated marker DYS385, locus specific analysis of DYS385a and b [19] was performed following the protocol published in [20].

### Population Genetics Analyses of Y-STR Data

For all calculations DYS389I repeat numbers were subtracted from the respective concatenated DYS389I/II alleles to assess the repeat count at DYS389b. Further, both DYS385 alleles were removed from the haplotypes unless explicitly stated otherwise. The repeat unit count based STR allele designations were converted to their respective inferred repeat block lengths, allowing for incomplete repeat units. Duplicated Y-STRs were treated as missing data in all computations. The Arlequin software (version 3.5.1.2) [21] was used to compute haplotype diversity (H) and *F*_ST_ distances from the Y-STR haplotypes. For calculation of Type I error probabilities (*P*), the number of permutations was set to 5,000 for pairwise genetic distances (*F*_ST_). To correct for multiple comparisons, the method described by [22] was used as suggested by [23]. The discrimination capacity (D) was calculated by dividing the number of different haplotypes by the total number of haplotypes found in the particular population (sub)sample.

GENALEX 6.4 [24] was used for computation of Shannon's mutual index for information (^S^ *H*_UA_) [25], principal coordinates analysis (PCA), and for spatial autocorrelation analysis [26]. For the latter, the “even sample sizes” option was activated. Both pairwise *F*_ST_ and ^S^ *H*_UA_ were used as measures for genetic distance between the East Tyrolean municipalities. Mountains can form virtually insuperable barriers for human movement. Thus, straight line distances are a poor measure for de facto geographical separation in an alpine landscape and a geographic distance matrix was constructed instead on the basis of present-day road distances between the blood donor's municipalities as determined with Google maps (http://maps.google.de). Under the assumption of *H*_0_ (lacking spatial structure), in the spatial autocorrelation analyses the number for permutations and bootstrap resampling was set to 9,999 to generate distributions of autocorrelation coefficients (*r*) by random shuffling.

Finally, the relationships between East Tyrol and a set of ten reference population samples at the haplotype level, PCA was performed on both pairwise *F*_ST_ and ^S^ *H*_UA_ distances.

### On-Line Data Access

All East Tyrolean Y-STR profiles and Y-SNP results obtained in this study were deposited in the Y Chromosome Haplotype Database [27], [28] (YHRD, http://www.yhrd.org) under the accession number YA003716 (East Tyrol, Austria [Tyrolean]). The accession numbers for the population samples from Salzburg (Austria, N = 203) and Upper Austria (Austria, N = 226) are YA003408 and YA003409, respectively.

The YHRD entries used as reference population samples can be found under the accession numbers: YA003601: West Croatia (N = 220); YA003595: North Croatia (N = 220) [29]; YA003463: Southern Poland (N = 380) [30]; YA003016: Central Portugal (N = 303) [31]; YA003474: Alpujarra de la Sierra (Spain, N = 50) [32]; YA003447: Modena (Italy-1, N = 130) [33]; YA003262: Ravenna (Italy-2, N = 384) [33]; and YA002890: North Tyrol (Austria, N = 135) [18].

## Results

### Onomastics

In East Tyrol the completion of the transition to an entirely Germanic speaking population took place before the utilization of surnames was custom in all social classes [34], [35]. As a result, the analysis of family names is unlikely to shed light on the early times of settlement. Accordingly, the examination of the blood-donors' family names revealed no names with Romance etymons. About 93% of the names had Germanic word roots and the remaining 7% had to be regarded as Bavarian names with a Slavic etymon as they were derived by the Bavarian speaking people from formerly Slavic place names (e. g. the Bavarian family name “Klaunzer“ is derived from the Slavic place name “Klaunz”, “Presslaber” from “Presslab”, “Ganzer” from “Ganz” and so on). Thus, all of the East Tyrolean blood-donors' surnames were adopted from the German speaking population. No further attempts were undertaken to relate the present-day Y-chromosomal diversity found in East Tyrol to surnames.

Rampl [5] reported that place names with a Romance or Slavic etymon are not evenly distributed over East Tyrol. Therefore, toponomastic analyses were performed.

**Figure 2:**
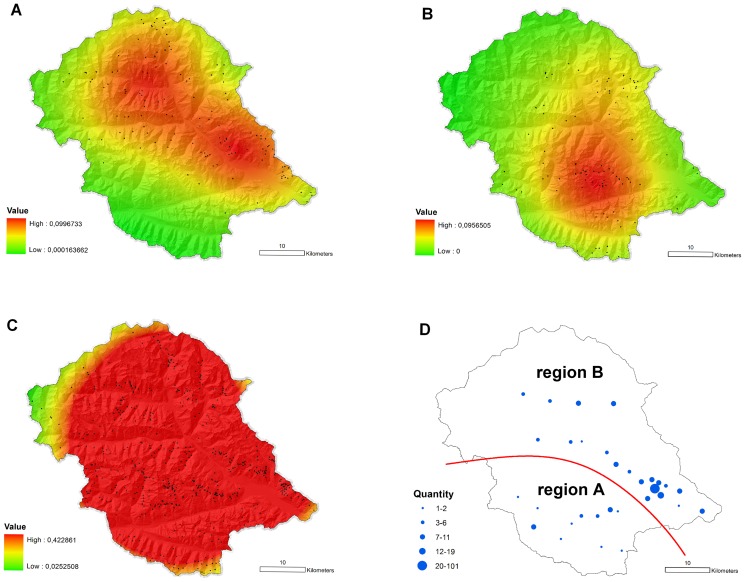
Spatial density distribution of pasture names in East Tyrol. The geographical distributions and frequencies of in total 853 pasture names with Slavic (panel **A**), Romance (panel **B**), and Germanic (panel **C**) etymons are shown. Black dots indicate the localization of the pastures. Panel **D** illustrates the localization of the blood donors' municipalities of birth and the subdivision of the study area into two regions of former Romance (A) respectively Slavic (B) main settlement.

From the 853 analyzed pasture names in East Tyrol 71% were derived from Germanic (Bavarian) etymons, 17% from Slavic etymons, and 12% from Romance etymons. While pasture names with Germanic etymons were evenly distributed in high density within the whole study area the names with Slavic etymons were spatially focused in the east and north of East Tyrol (Fig. 2). Geographically, these are the lower Drau, Isel, Kals, Virgen and the Defereggen valleys (Fig. 1). No names with Slavic etymons were found in the southwestern Puster valley (Fig. 2). The pasture names with Romance etymons focus mainly in the southern part of East Tyrol (Gail, Puster, and Villgraten valley, Fig. 2). The slight northeastward trend observed in the distribution of Romance etymons is solely caused by the Kals valley, a medieval Romance linguistic enclave, which was separated from the Romance main territory in the 10^th^ century [36]. On the basis of these results, East Tyrol was divided into two regions of former Romance (Puster, Gail, and Villgraten valley; region A) and Slavic (Isel, lower Drau, Defereggen, Virgen, and Kals valley; region B) main settlement (Fig. 2).

### Descriptive Sample Statistics

During a single blood collection campaign in the city of Lienz, 287 samples from voluntary East Tyrolean donors were obtained. Groups of samples featuring both identical Y-SNP and Y-STR results as well as shared paternal descent (grandfather/father as determined from surnames, date of birth, and place of birth/residence) were considered to derive from close paternal relatives. In all such cases, only one specimen per group was retained in the sample set. Consequently, the final population sample comprised 270 specimens from, according to this filter, paternally unrelated men. Although the sample collection took place only in the district's capital, 29 out of the 33 East Tyrolean municipalities were covered (Table S5). The two largest contributions to the sample pool originated from Lienz (99 specimens corresponding to ∼0.8% of Lienz's total population) and Tristach (19 samples, ∼1.4%). On average, ∼0.7% of a covered municipality's total population was sampled (Table S5). In summary, 35 specimens (13.0%) were assigned to region A and 235 (87.0%) fell into region B.

One-hundred and forty-one samples (52.2%) were donated by probands who declared East Tyrolean residence dating back to their paternal grandfathers. Within this group, a high rate of patrilocality was observed. In region A (B), 23 (103) individuals could be traced back to paternal grandfathers coming from the same region, and in 2 (13) instances either the grandfather or the father came from region B (A).

For 59 donors (21.9%), insufficient personal information was available to assess third generation East Tyrolean residence. However, 80% of them (n = 47) specified that their fathers were from East Tyrol. The remaining 70 samples (25.9%; region A: 1 proband; region B: 69 probands) came from donors descending from grandfathers/fathers with non East Tyrolean provenance. Three quarters of these immigrants stemmed either from North Tyrol (n = 7) or regions adjacent to East Tyrol (Carinthia: 28; South Tyrol: 10; Salzburg: 7; Veneto: 1). Fourteen blood donors located their grandfathers/fathers in other parts of Austria, in two cases the fathers came from Germany, and the father of one proband emigrated from Belarus.

Eighty-three (45.4%) of the 183 subjects with migration records covering three generations reported unchanged locality since their paternal grandfathers, whereas in 84 (45.9%) and 16 (8.7%) cases one and two migrational events, respectively, were documented. In general, the total grandfather-father-proband migratory distances were low, with a median value amounting to 5 km (26 probands from region A: 0 km; 157 probands from region B: 11 km; Fig. S2). For comparison, the average pairwise distances between municipalities in East Tyrol or within regions A and B amounted to 36 km, 20 km, and 24 km, respectively (Table S6).

Typical mutation rates for the Y-STR markers used in this study are in the range of 2×10^−3^ per generation or lower [28], [37], [38]. Thus, assuming that the probands' Y chromosomes most closely matched their fathers' and grandfathers' appeared valid. This allowed the analysis of the Y-chromosomal landscape present in the late 19^th^ and early to mid 20^th^ century East Tyrol, as the average year of birth of the fathers and paternal grandfathers was 1925 and 1890, respectively (Fig. S3).

Under the two and three generations of residence criteria 205 (75.9%) and 141 (52.2%) data sets, respectively, remained in the analysis. On the basis of information regarding the fathers' and grandfathers' places of birth/residence, both of these sub-samples split up in about one quarter and three quarters to regions A and B, respectively (Table S5).

### Y-SNP Data

**Figure 3:**
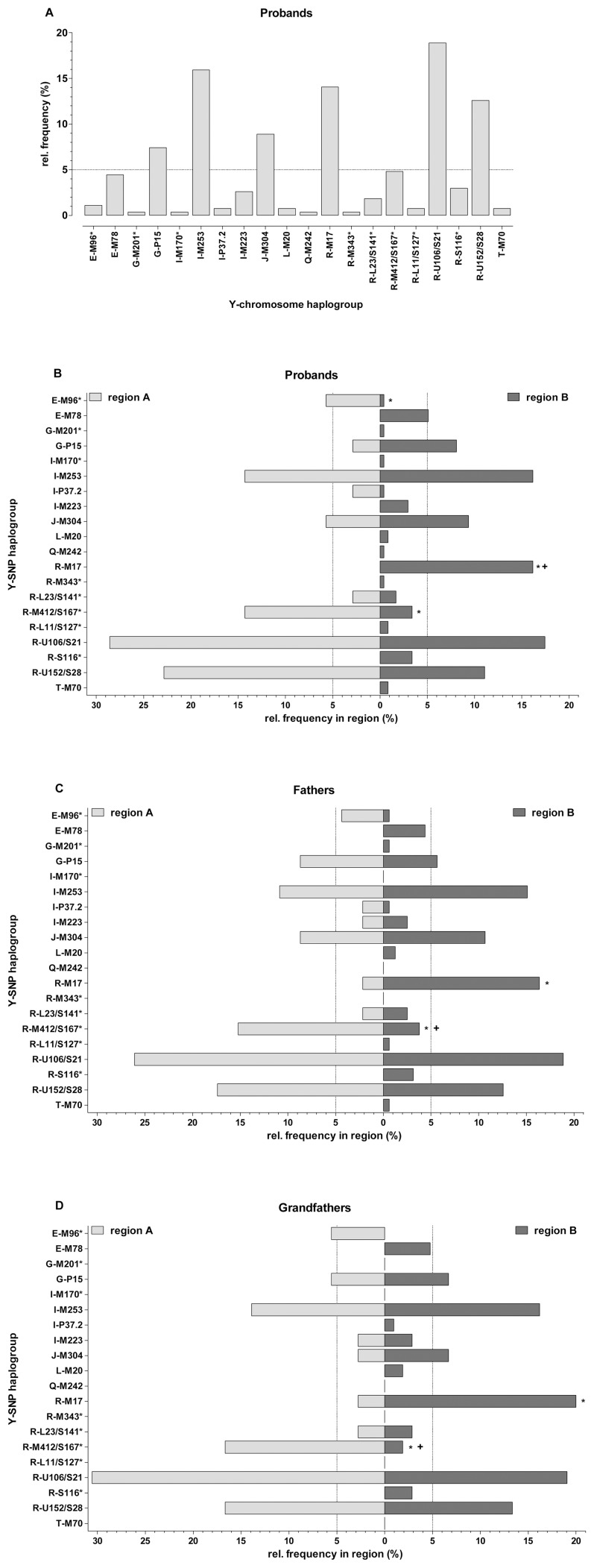
Y-chromosomal haplogroup frequencies. Relative haplogroup frequencies found for the Y-chromosomes comprised in the entire East Tyrolean population sample (panel **A**) or the two sub-samples from regions A and B (panel **B**). Panels **C** and **D** depict the results obtained under a two and three generations of residence criterion for data analysis. The probands' Y-SNP profiles were assigned both to their fathers' (panel **C**) and paternal grandfathers' (panel **D**) municipalities of birth/residence. Asterisks at the top of bars indicate *P* values≤0.05 (Fisher's exact test) and “+” designates statistical significance after correction for multiple comparisons using the method described by [22]. All *P* values obtained for the pairwise haplogroup frequency differences between regions A and B exceeded the critical value of the Bonferroni adjustment for multiple testing [58].

By typing a set of 27 Y-chromosomal SNP loci, the 270 samples in this study were assigned to 20 of the 30 theoretically distinguishable haplo/paragroups (Fig. 3, Fig. S1, Table S1, Table S7, Table S8). The major proportion of Y chromosomes (n = 152, 56.3%) was found to belong to haplogroup R-M173 (R1), followed by haplogroups I-M253 (I1; n = 43, 15.9%), J-M304 (J; n = 24, 8.9%), and G-P15 (G2a; n = 20, 7.4%). All other haplogroups showed population frequencies of less than 5% in the combined East Tyrolean sample (Fig. 3). The haplogroup R-M173 derived chromosomes comprised two major subgroups. One quarter of these samples (n = 38) could be assigned to haplogroup R-M17 (R1a1a) and the remaining three quarters (n = 114) fell into the R-M343 (R1b) clade. Inside the R1b cohort, haplogroups R-M412/S167* (n = 13), R-U106/S21 (n = 51), and R-U152/S28 (n = 34) accounted for 87.7% of the Y chromosomes.

Splitting the East Tyrolean population sample into regions A and B resulted in a partitioning of haplogroups E-M78, R-M17, R-M412/S167*, and R-S116*. E-M78, R-M17 and R-S116* Y chromosomes were exclusively found in region B whereas samples assigned to R-M412/S167*, R-U106/S21, and R-U152/S28 reached higher frequencies in region A (Fig. 3, Table S7). When attributing the samples to the fathers' and grandfathers' places of birth/residence, as reported by the participants, practically identical patterns were obtained for most of the haplogroups (Fig. 3).

Y chromosomes belonging to haplogroups G-P15, I-M253, and J-M304 showed much lower regionalization in their frequencies (Fig. 3) at all three generation levels.

### Y-STR Data

For all 270 specimens in the East Tyrolean population sample, full 17-locus Y-STR haplotypes were obtained (Table S8). Basic measures for molecular diversity (haplotype diversity (H), discrimination capacity (D), mean number of pairwise different alleles) were calculated for all generational levels under different grouping schemes (i.e. entire East Tyrolean sample, region A and B sub-samples). By inclusion of the DYS385 phenotypes, H and D were in the range of ∼99% and >80%, respectively, regardless of grouping scheme and generational level (Table S9).

The geographic distribution of Y-STR haplotypes was investigated by spatial autocorrelation analysis to compute a correlation coefficient *r* between geographical (road-distance) and genetic (*F*_ST_, ^S^ *H*_UA_) distances. Testing the overall statistical significance of the spatial autocorrelograms [26] gave *P* values of 0.086 and 0.006 for *F*_ST_ and ^S^ *H*_UA_ population statistics, respectively. However, both the correlograms obtained with *F*_ST_ and ^S^ *H*_UA_ distances did not yield information in favor of spatial genetic structuring of the East Tyrolean data set (Fig. S4).

Inspection of the haplotype variability within the three major R1b sub-haplogroups revealed a marked difference in the mean number of pairwise different alleles among these lineages. This trend, however, was seen in both regions. R-M412/S167* chromosomes exhibited on average 2.2±1.1 (n = 10 pairwise comparisons) and 1.9±1.6 (n = 28) different alleles per 17-locus haplotype in regions A and B, respectively (*P*>0.05). R-U106/S21 chromosomes showed mean values of 5.7±2.9 (n = 45, region A) and 6.9±1.7 (n = 820, region B) variant repeat counts (*P*<0.0001). Finally, for haplogroup R-152/S28 these figures amounted to 5.5±1.5 (n = 28) and 5.8±2.1 (n = 325) in regions A and B, respectively (*P*>0.05). De facto equivalent results were obtained for the Y chromosomes in those haplogroups when analyzing the number of multi-step neighbor alleles per pairwise 17-locus haplotype comparison. The values for R-M412/S167*, R-U106/S21, and R-U152/S28 were A: 0.0±0.0 (n = 10)/B: 0.7±0.6 (n = 28; *P*>0.05), A: 1.8±0.9 (n = 45)/B: 2.3±1.0 (n = 820; *P*<0.01), and A: 1.6±0.8 (n = 28)/B: 2.0±1.0 (n = 325; *P*>0.05), respectively.

We further computed pairwise genetic distances (*F*_ST_) on the basis of the Y-STR haplotypes obtained for the East Tyrolean sample and a set of ten reference populations. The latter were grouped with regard to their language-family memberships (Germanic: Salzburg, Upper Austria, North Tyrol; Romance: Italy-1 (Modena), Italy-2 (Ravenna), Spain, Portugal; Slavic: North Croatia, West Croatia, South Poland).

According to the *P* values that we obtained for the *F*_ST_ distances, East Tyrol grouped at the Y-STR haplotype level with the entire sample-set forming the Germanic language reference group by featuring statistically insignificant *F*_ST_ values, whereas it clearly contrasted with the Romance and the Slavic reference samples (Table S10).

After splitting the East Tyrolean population sample, *F*_ST_ *P* values suggested for region A Y chromosomes proximity to the Romance speaking reference populations, whereas for the region B haplotypes insignificant *F*_ST_ values were obtained only with North Tyrol, Salzburg, and Upper Austria. This patterning was seen at all three generational levels of sample placement (Table S10).

**Figure 4:**
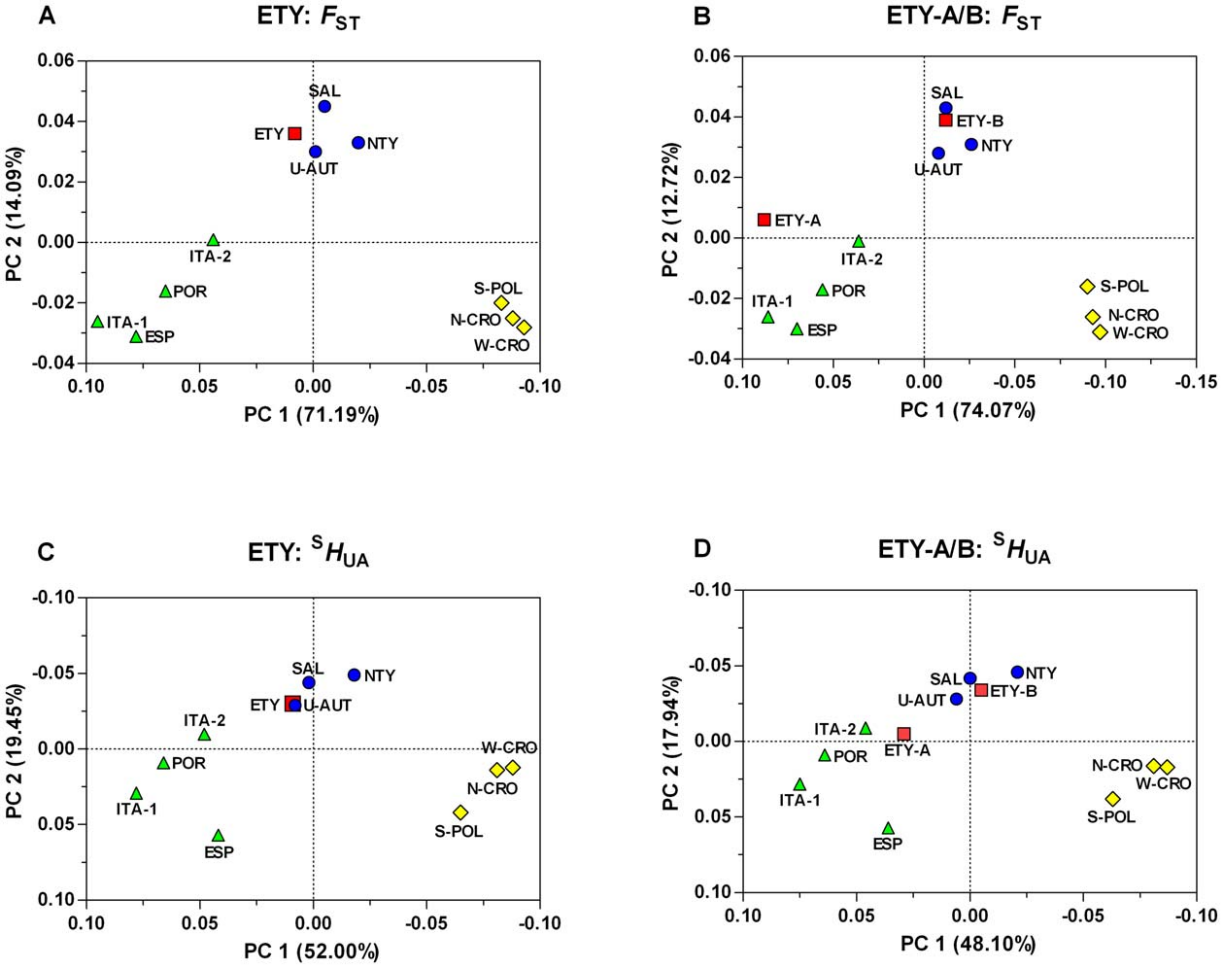
Principal coordinates analysis at the probands-level. Plots showing the first and second principal coordinates determined by PCA using both pairwise *F*_ST_ (panels **A**, **B**) and ^S^ *H*_UA_ (panels **C**, **D**) distances, as obtained for the Y-STR haplotypes comprised in the combined and subdivided East Tyrolean population sample and a set of ten reference datasets. For analyses, the East Tyrolean profiles were assigned to the probands' places of birth/residence. **ETY**: East Tyrol, **ETY-A**: East Tyrol region A, **ETY-B**: East Tyrol region B. Reference populations: **SAL**: Salzburg (Austria), **U-AUT**: Upper Austria (Austria), **ESP**: Spain, **ITA-1**: North Italy (Modena), **ITA-2**: North Italy (Ravenna), **N-CRO**: North Croatia, **NTY**: North Tyrol, **S**-**POL**: South Poland, **POR**: Portugal, **W-CRO**: West Croatia. Green triangles, blue circles and yellow diamonds indicate Romance, Germanic and Slavic language family membership of the reference populations, respectively.

To display and quantify the major patterning in the Y-STR data, the relationships among the pairwise *F*_ST_ values were plotted by means of PCA for all three levels of generational sample assignment (Fig. 4, Fig. S5, Fig. S6). About 85% of the total haplotypic variation could be attributed to the first (∼71%) and second (∼14%) principal coordinates. The reference populations clustered by language along principal coordinate 1 and the East Tyrolean Y chromosomes appeared at a position close to the reference populations from the Germanic speaking group (Fig. 4). Notably, the samples from region A clearly clustered with the Romance reference populations and the Y chromosomes from region B moved even closer to North Tyrol, Salzburg, and Upper Austria (Fig. 4). Again, this picture remained largely unaffected by locating the East Tyrolean Y-STR haplotypes at their fathers' and grandfathers' places of birth/residence (Fig. S5, Fig. S6).

When using ^S^ *H*_UA_ as an alternative measure for population differentiation instead of *F*_ST_ values, de facto equivalent results were obtained with PCA (Fig. 4, Fig S5, Fig. S6).

## Discussion

Alpine landscapes form highly structured substrates for settlement and occupation. This holds particularly true in agrarian-oriented societies relying on alpine farming in a seasonal fashion. Yet, in East Tyrol the picture becomes even more complex, as for several centuries members of the Romance, the Slavic and the Germanic language families settled contemporaneously in East Tyrol during its early history. Present-day East Tyrol, however, is inhabited by an exclusively German speaking population, a state that lasted for no less than the last 600 years.

Onomastic analysis of the surnames of the blood donors participating in this study failed to echo the early settlement of members of all three major European language groups in East Tyrol. According to our results, all of the probands' surnames had to be regarded as being adopted from the Germanic (Bavarian) language group. Hence, any genetic investigation relating to surnames would have failed to yield meaningful results concerning the early time of settlement. Place names (in the broadest meaning), however, root deeper in time as they are usually given at the beginning of a colonization process due to the need to name the new, unnamed territory. In general, one can assume a direct correlation between duration and density of a settlement with the number of place names. Thus, the density of place names originating in a particular language layer is an indicator of the intensity and duration of the settling by the people speaking this language. Especially, the names of pastures show a good balance of different processes of naming as they are derived from settlement names, farmyard names, field names, mountain names, and so on. Hence, they reflect both oikonyms (settlement names) and toponyms (here field names in a broad sense) and represent an ideal basis to conduct distributional research in alpine regions.

Due to the fact that East Tyrol has been an entirely German speaking region for centuries, pasture names with Germanic etymons were found to be evenly distributed in high density within the whole study area (Fig. 2). In contrast to this finding, pasture names with Slavic and Romance etymons showed clear and complementary spatial structuring in their density distributions. To test if this deep rooting segmentation still correlates with present day Y chromosome diversity in East Tyrol, the study area was divided, on the basis of these results, into two regions (Fig. 2): one southwestern zone of former Roman main settlement (region A) and one bigger zone (region B), which covered the former Slavic main area of settlement.

To gain an understanding of the latter, both a set of 27 phylogenetically informative binary Y-SNPs and 17 polymorphic microsatellite markers were investigated. With the exception of the two pseudoautosomal regions, the major part of the exclusively paternally transmitted human Y chromosome does not undergo recombination. This results in stable Y-SNP haplogroups and informative Y-STR haplotypes. Further, the two marker types exhibit largely differing mutation rates (e.g. [39], [40]). Consequently, Y-SNPs and Y-STRs cover human population history at different time scales [41], [42].

Mutations generating biallelic SNPs are usually considered to be unique events in the Y-chromosomal phylogeny, which makes them ideal candidates for the analysis of events dating deeply back in human history and evolution [43]. When investigated at the level of these slowly evolving markers, the Y-chromosomal landscape in Europe is largely shaped by the markedly differing and complementary dispersal patterns of two major clades R1a and R1b (R-M343) [44], [45]. Haplogroup R1b reaches its modal frequency in the west and north-west of Europe and shows a decline toward the east and the Balkans, whereas Y chromosomes belonging to haplogroup R1a and here especially to the dominant R-M17 (R1a1a) lineage complement this picture by reaching the highest frequencies in the east of the continent [44]–[50]. For instance, in the Iberian Peninsula, in France, West Germany, and North Italy, the dominant R-M269 branch of the European R-M343 (R1b) Y chromosomes (Fig. S1) reaches population frequencies of 50%–60%, whereas it is encountered in West Austria (North Tyrol), East Germany and East Poland at frequencies amounting to about 32%, 38% and 13%, respectively [18], [51], [52]. Moreover, Myres and coworkers [51] recently demonstrated that it is particularly the group of M412/S167 derived Y chromosomes that predominates in West and Central/North Europe. Branches R-U106/S21 and R-U152/S28, both nested in R-M412/S167 (Fig. S1), reach modal frequencies in North/Central Europe and North Italy/France, respectively [51]–[53], whereas the major S116 subclade is mainly found in western parts of Europe, where it can reach frequencies >70% [51].

The western border of the geographic expansion of haplogroup R-M17 Y chromosomes is to be found in Central Europe and largely follows the political border separating present-day Poland (57%) and Germany (East: ∼30%, South: ∼14%, West: ∼10%) [42]. Frequencies of about 15% and 10% were also found for Austria [18] and North-East Italy [48], respectively. In South Italy and in West Europe R-M17 chromosomes are not present at informative frequencies.

In this study, the proportion of Y chromosomes carrying the derived M17 allele was 14.1%, a value that nearly perfectly matched those reported for West Austria (North Tyrol, 15.4%) and South Germany (Munich; 14.3%) [18], [42]. However, haplogroup R-M17 was completely absent in the East Tyrolean sub-sample from region A, but made up to 16% in region B. This result remained practically unchanged when assigning the probands to their respective fathers' or grandfathers' places of birth/residence (Fig. 3).

Haplogroup R-M343 (R1b) accounted for 42.2% of all Y chromosomes in our population sample, a value that was markedly higher than that determined for North Tyrol (∼32%) [18]. Even more pronounced differences became evident when analyzing regions A and B separately. In regions A and B haplogroup R-M343 reached frequencies of 68.6% and 37.9%, respectively. This contrast was statistically significant (*P* = 0.0008) and placed the region A sub-sample closer to Romance speaking populations in Western and Central Europe as well as North Italy than to e.g. North Tyrol. The opposite applied to the sub-sample from region B.

At a higher haplogroup resolution, the distribution pattern within R-M343 became more complex. The major subclades R-U106/S21 (18.9%) and R-U152/S28 (12.6%) were detected at frequencies that were almost identical to those reported for Germany (20.8% and 11.7%, respectively) [52]. At the probands level, the R-S116 sub-haplogroup R-U152/S28 reached in region A a two times higher frequency than in region B (A: ∼23%, B: ∼11%). This contrast, however, was diminished when placing the samples to the fathers and grandfathers places of birth/residence (Fig. 3, Table S7). The region A∶B ratio determined for haplogroup R-U106/S21 was in the range of 1.4–1.6 for all generational levels of analysis. None of these differences proved statistically significant (Table S7).

Haplogroup R-M412/S167* was found at low frequencies in the combined East Tyrolean sample. However, the R-M412/S167* chromosomes were sorted by the subdivision of the study area and reached in region A levels of ∼14% whereas their frequency in region B was well below the 5% threshold. At the probands and fathers level of analysis region A featured approximately fourfold higher frequencies of these chromosomes than region B. This ratio changed to about nine when placing the samples at the grandfathers' places of birth/residence. These contrasts remained statistically significant after correcting for multiple comparisons [22] at the fathers and grandfathers analysis level.

Haplogroups E-M96* and E-M78 were found predominantly in regions A and B, respectively, albeit at low frequencies (Fig. 3, Table S7). The significance of these findings for an understanding of the phylogeographic variation of human Y chromosomes in East Tyrol remains unclear.

Y chromosomes exhibiting the derived base state at the M253 locus constituted one of the major groups in East Tyrol. Haplogroup I-M253 (I1) chromosomes are known to display a gradient with a pronounced decrease in frequency from North Europe toward South-East Europe [54] and Kayser and coworkers [42] reported a four times higher frequency of I1 chromosomes in Germany (25%) as compared to Poland (∼6%). In this study, 15.9% (region A: 14.3%, region B: 16.7%) of the East Tyrolean samples could be assigned to haplogroup I-M253. Obviously, this balanced frequency distribution did not provide easy to interpret information in respect of our attempt to shed light on the significance of historical events on the present-day Y chromosome variation in East Tyrol.

The remaining haplogroups identified in this study occurred at non-informative population frequencies <5% and showed no clear distribution pattern between regions A and B.

Y-STRs are evolving at a much higher rate than Y-SNPs and are therefore considered the better choice for the population genetic investigation of more recent historical events [41], [42].

In an initial attempt to test for spatial genetic structuring in the distribution of the East Tyrolean Y-STR haplotypes, a spatial autocorrelation analysis [24], [26] was performed using present-day road distances as geographic and *F*_ST_ as well as ^S^ *H*_UA_ as genetic distance measures. Between some of the East Tyrolean municipalities statistically significant pairwise *F*_ST_ distances were detected (Table S11). This picture, however, was probably obscured by the low number of individuals sampled for the majority of municipalities. Furthermore, statistical testing indicated significant “non-flatness” for the correlograms obtained with ^S^ *H*_UA_. However, for both genetic distance measures visual inspection of the correlograms failed to provide evidence for non-random spatial genetic patterning (Fig. S4). Hence, in contrast to the Y-SNP haplogroup data this haplotype based approach did not yield convincing data in favor of a segmentation of East Tyrol's present-day Y-chromosomal landscape.

Nevertheless, additional attempts were undertaken at the Y-STR haplotype level to test for a potential geographic segmentation pattern of East Tyrol's Y chromosomes that still might bear an imprint of East Tyrol's early history.

Aside from being convenient, the use of a commercially available and widely used kit for Y-STR typing made it easier to find suitable haplotype data sets for comparisons as well. Here we used haplotypic data from Portugal, Spain, North Italy (Modena, Ravenna), North and West Croatia, South Poland, and Austria (North Tyrol, Salzburg, Upper Austria) as reference population samples for further analyses. These reference samples were categorized according to their language family memberships (Slavic: North Croatia, West Croatia, South Poland; Romance: Italy-1 (Modena), Italy-2 (Ravenna), Spain, Portugal; Germanic: Salzburg, Upper Austria, North Tyrol).

Pairwise *F*_ST_ and ^S^ *H*_UA_ distances between the East Tyrolean sample, which comprised either data from all probands or only from donors who provided sufficient information for locating their profiles at the fathers' or grandfathers' places of birth/residence, and the ten reference population samples were computed on the basis of Y-STR haplotypes.

Principal coordinates analysis was performed to map the relationships among the genetic distances. The reference populations clearly clustered by language along principal coordinate 1 and the combined East Tyrolean data set closely matched with the Germanic group (Fig. 4). Analysis of the partitioned East Tyrolean sample, however, allocated the region A subsample to the Romance speaking populations and the region B Y chromosomes to the Germanic cluster, regardless of the applied distance measure (Fig. 4). Practically equivalent results were obtained when locating the probands' haplotypes at their fathers' and grandfathers' places of birth/residence (Fig. S5, Fig. S6).

The patterning seen with PCA was supported by the Type I error probabilities of the underlying pairwise *F*_ST_ distances (Table S10) and clearly corroborated the coarse picture drawn by the Y-SNP haplogroup frequency distributions.

Subdividing the East Tyrolean sample showed an astonishingly clear effect on the subsample from the former Romance main territory (region A). The small sample size (n = 35) could have accounted for this finding. However, a major part of the Y-SNP signal that differentiated the two subsets from regions A and B came from haplogroup R-M17, which constituted one of the most frequent clades in East Tyrol. Hence, an overrepresentation of R-M17 chromosomes should be expected for the small sample from region A rather than their complete lack in this area. However, too optimistic population frequency estimates for haplogroup R-M343 and its subgroups can not be fully excluded for region A. Yet, the overall frequency of R1b chromosomes appeared too high to be solely explained by random ascertainment error.

Marked genetic drift due to a small effective population size is a factor that is known to cause population differentiation. Thus, we set out to roughly assess its significance for the Y-chromosomal distribution pattern we observed in region A. The respective relative frequencies of the major lineages R-M17, R-U106/S21, R156/S28, and the concatenated R-M343 clade did not differ statistically significant among the three valleys in region A (*P*≥0.29 for all comparisons, Fisher's exact test). The sample numbers were particularly low for the Gail and the Villgraten valleys. Therefore, this approach allowed only for the exclusion of a pronounced effect of genetic drift acting differently in the three valleys.

The standard genetic diversity indices obtained for the Y-STR haplotypes from region A (e.g. D = 100%, Table S9) obviously pointed against an overall low Y-chromosomal diversity in region A, which might have been an indication for a significant role of genetic drift. Furthermore, the pairwise *F*_ST_ distances between the 15-locus Y-STR haplotypes from the Puster, Gail, and Villgraten valleys were not statistically significant.

On average, ∼11 pairwise different Y-STR alleles were found for the combined as well as sub-divided population sample from East Tyrol (Table S9). However, a more differentiated picture was gained by the analysis of the differences in allelic variability among the three major R1b sub-haplogroups. R-M412 exhibited on average in both regions only about 2 different Y-STR alleles per 17-locus haplotype. For haplogroup R-U106/S21 and R-U152/S28 chromosomes these values were about three times higher. However, the differences between the two regions were rather low, albeit statistically significant in the case of R-U106/S21. An equivalent picture was obtained for these three R1b subgroups when looking at of the average number of multiple-step neighbors per pairwise haplotype comparison. Again, the differences between the two East Tyrolean sub-samples were small.

On the basis of these data, we can not fully exclude ascertainment bias and/or genetic drift as “shaping forces” for our results. However, neither of them convincingly explained the marked geospatial structuring of modern Y chromosomes. Therefore, we suggest that the question of whether or not the segmentation of East Tyrol during its early history of settlement, as deduced by the frequency distribution of the Romance and Slavic etymons of pasture names, still correlates with the Y-chromosomal landscape in present-day East Tyrol, can be positively answered for the region of ancient Roman main settlement (region A), even centuries after the Romance language was completely replaced by the Bavarian dialect brought to East Tyrol by the Germanic speaking people. For Y chromosomes from region B (area of ancient Slavic main settlement), however, no indication for a clustering with the Slavic speaking reference populations was detected.

Beyond these findings, the results obtained for the Y chromosomes sampled in region A, in particular, demand an explanation. Does the lack of haplogroup R-M17 still relate to the outcome of the battle of Aguntum in the year 610, which stopped the westbound Slavic settlement in East Tyrol [1]? How can we explain the striking clustering of the haplotypes from region A with the reference samples from Romance speaking populations? The clear phylogeographic differentiation between regions A and B takes place at the microgeographic scale. For instance, the average pairwise road distances among municipalities in the lower Drau, Virgen, and Defereggen valley (all region B), and in the Puster and Gail valley (both region A) amount to about 40 km and 22 km, respectively (Table S6).

Onomastic analysis of the probands' family names revealed no indication of significant immigration to East Tyrol from the Romance speaking neighbouring areas since the common usage of surnames in the 14^th^ century. This is corroborated by the high degree of patrilocality that can be deduced from the personal information regarding the fathers and paternal grandfathers provided by the blood donors and dating back to the 19^th^ century. Thus, significant migrational events that took place within the last 200 years can be excluded as an explanation for our findings. The phylogeographic signal therefore must root deeper in time. Demographic conditions limiting the effective population size are known to have been a factor in Tyrol until the beginning of the 20^th^ century. For instance, in the 1830's the average annual rate of marriages was about 0.7%. In 1820, tight restrictions became effective that precluded all persons without a secure income from marriage. This regulation was particularly strict for non-residents. As a consequence, the average annual number of marriages per 1000 residents dropped to less than five until 1880 [55]. Due to an exceptionally low illegitimacy rate of about 0.5%, it was basically only wealthy people (e.g. land owners) who contributed to the next generation. The typical age at marriage was high for both sexes and both the annual birth rates and the infant mortality rates were relatively low in Tyrol [55]. In the 19^th^ century the birth rates approximated over time the mortality rate, which caused, in combination with a latent outflow of people, a reduction in population growth rates. In the 1820's, for instance, 33% more births than deaths were reported per year. This figure amounted to 17% between 1860–1870 and dropped to less than 1% until 1880 [55], [56]. The reversal of this trend coincided with the discontinuation of the marriage restrictions at the beginning of the 20^th^ century and East Tyrol's connection to the “Drautal” railroad at the end of the 19^th^ century, which fostered economical growth and created new fields of occupation.

The sparse historical facts covering the early times of settlement in East Tyrol and later on, allow us only to speculate about the demographic processes that shaped the Y-chromosomal landscape in East Tyrol. However, linguistic science provides strong support for a long-term seclusion of the East Tyrolean area, since the archaic structure of the indigenous vernacular reflects only little influence from the surrounding dialects [57].

It seems reasonable to assume that in a small, agriculturally dominated alpine area, philopatric men (e.g. land owners) have contributed substantially to the Y-chromosomal pool, thus preserving in region A Y chromosomes dating back to the early Romance speaking settlers.

Our results suggest cultural diffusion rather than population replacement as the underlying mechanism for the complete transition from a Romance to a Germanic speaking population in the southwestern part of East Tyrol.

Further, the outcomes of this study formidably demonstrate how largely differing disciplines can complement each other. Here, the toponomastic analysis of pasture names provided essential and otherwise unavailable information for the detection, analysis, and interpretation of a marked substructuring in a population of human Y chromosomes initially considered homogeneous.

## Supporting Information

Figure S1 **Binary Y-SNP haplogroup tree.** Phylogenetic tree depicting the relationships among the binary Y-chromosomal haplogroups defined by the 27 SNPs analyzed in this study. Markers that were typed by Sanger sequencing instead of a multiplexed PCR/single nucleotide primer extension assay are indicated by italicised bold typeface SNP designations.
(TIF)

Figure S2 **Intergenerational migration distances.** Father-proband (panel **A**), grandfather-proband (panel **B**), grandfather-father (panel **C**), and grandfather-father-proband (panel **D**) migration distances were determined as present-day road distances between the probands', their fathers', and paternal grandfathers' places of birth/residence. **ETA**: East Tyrol region A; **ETB**: East Tyrol region B; **ETY**: East Tyrol.
(TIF)

Figure S3 **Year of birth statistics.** Box-and-Whiskers plots summarizing the probands', their fathers', and paternal grandfathers' year of birth statistics. The whiskers depict the lowest (highest) datum falling within the 1.5×(Q3-Q1) interquartile range below (above) the first (third) quartile. Data points outside the 1.5 interquartile ranges are shown as dots.
(TIF)

Figure S4 **Spatial autocorrelation analysis.** Correlograms plotting the spatial autocorrelation coefficient (*r*) as a function of geographical distance class. Dashed lines indicate the upper and lower bounds of the 95% confidence belt for *r* as obtained by random shuffling. Spatial structuring is inferred when the measured *r* value for a particular distance class falls outside this belt. Both *F*_ST_ (panel **A**) and ^S^ *H*_UA_ (panel **B**) distances were used as measures for genetic differentiation. Geographical distances represent present-day road distances between municipalities.
(TIF)

Figure S5 **Principal coordinates analysis at the fathers-level.** Plots showing the first and second principal coordinates determined by PCA using both pairwise *F*_ST_ (panels **A**, **B**) and ^S^ *H*_UA_ (panels **C**, **D**) distances, as obtained for the Y-STR haplotypes comprised in the combined and subdivided East Tyrolean population sample and a set of ten reference datasets. For analyses, the East Tyrolean profiles were assigned to the fathers' places of birth/residence, as reported by the probands. **ETY**: East Tyrol, **ETY-A**: East Tyrol region A, **ETY-B**: East Tyrol region B. Reference populations: **SAL**: Salzburg (Austria), **U-AUT**: Upper Austria (Austria), **ESP**: Spain, **ITA-1**: North Italy (Modena), **ITA-2**: North Italy (Ravenna), **N-CRO**: North Croatia, **NTY**: North Tyrol, **S**-**POL**: South Poland, **POR**: Portugal, **W-CRO**: West Croatia. Green triangles, blue circles and yellow diamonds indicate Romance, Germanic and Slavic language family membership of the reference populations, respectively.
(TIF)

Figure S6 **Principal coordinates analysis at the grandfathers-level.** Plots showing the first and second principal coordinates determined by PCA using both pairwise *F*_ST_ (panels **A**, **B**) and ^S^ *H*_UA_ (panels **C**, **D**) distances, as obtained for the Y-STR haplotypes comprised in the combined and subdivided East Tyrolean population sample and a set of ten reference datasets. For analyses, the East Tyrolean profiles were assigned to the paternal grandfathers' places of birth/residence, as reported by the probands. **ETY**: East Tyrol, **ETY-A**: East Tyrol region A, **ETY-B**: East Tyrol region B. Reference populations: **SAL**: Salzburg (Austria), **U-AUT**: Upper Austria (Austria), **ESP**: Spain, **ITA-1**: North Italy (Modena), **ITA-2**: North Italy (Ravenna), **N-CRO**: North Croatia, **NTY**: North Tyrol, **S**-**POL**: South Poland, **POR**: Portugal, **W-CRO**: West Croatia. Green triangles, blue circles and yellow diamonds indicate Romance, Germanic and Slavic language family membership of the reference populations, respectively.
(TIF)

Table S1 **Y-SNP marker related information.** Supporting information regarding the binary Y-chromosomal markers analyzed in this study. Haplogroup designations were taken from the literature [12], [43], [51]–[53], [59]–[64] and the ISOGG 2012 tree (International Society of Genetic Genealogy 2012, http://www.isogg.org/tree/index.html).
(XLS)

Table S2 **Amplification/sequencing primers.** The sequences of the primers used for PCR amplification and sequencing were either taken from the literature [51], [65]–[69] or designed in-house.
(DOC)

Table S3 **Oligonucleotides used for 19-plex single nucleotide primer extension.** The primer sequences were either taken from the literature [65]–[68], [70] or designed in-house.
(DOC)

Table S4 **Amplicon sequences.** Overview of the sequences of all PCR products generated for genotyping the haplogroup diagnostic Y-SNPs [11], [51], [59], [60], [71]–[80]. All sequences are shown in Y chromosome+strand orientation. PCR primer binding regions are depicted by upper case, bold face letters, those for single base extension primers are underlined. SNP positions are shown in parentheses.
(DOC)

Table S5 **Sampling statistics.** Sampling coverage and geographic localizations of the East Tyrolean municipalities are shown.
(XLS)

Table S6 **Average road distances.** Average pairwise road-distances among all municipalities in the respective East Tyrolean regions and valleys are shown.
(XLS)

Table S7 **Y-SNP haplogroup frequencies.** The absolute and relative Y-chromosomal SNP haplogroup frequencies that were obtained for the East Tyrolean population sample and its sub-samples from regions A and B are shown. For analyses under the two and three generations of residence criterion, the probands' Y-SNP profiles were allocated to the fathers' and paternal grandfathers' places of birth/residence, respectively. Confidence intervals for single proportions were calculated using the modified Wald method [81] as implemented in the GraphPad QuickCalcs (GraphPad Software, La Jolla, CA, USA; http://www.graphpad.com/quickcalcs/CatMenu.cfm). For attributing statistical significance, a critical *P* value of α = 0.05 was used. Asterisks indicate statistical significance after correcting for multiple testing using the method described by [22]. The critical value for the Bonferroni adjustment of α for multiplicity was calculated according to [58].
(XLS)

Table S8 **Y-STR profiles and Y-SNP haplogroup assignments.** The regions of birth/residence, the 17-locus Y-STR haplotypes, and the Y-SNP haplogroup assignments are shown for all probands.
(XLS)

Table S9 **Standard diversity indices for the Y-chromosomal 17 locus Y-STR haplotypes.** For analyses under the two and three generations of residence criterion, the probands' profiles were allocated to the fathers' and paternal grandfathers' places of birth/residence, respectively.
(XLS)

Table S10 **Pairwise** ***F*** **_ST_ and** ***P*** **Values.** Pairwise comparisons between the East Tyrolean and ten reference population samples at the haplotypic level. *F*_ST_ values, as computed with Arlequin, are shown below and *P* values (5,040 permutations) above he diagonal. For analyses under the two and three generations of residence criterion, the probands' profiles were allocated to the fathers' and paternal grandfathers' places of birth/residence, respectively. **ETY**: East Tyrol, **ETY-A**: East Tyrol region A, **ETY-B**: East Tyrol region B. Reference populations: **SAL**: Salzburg (Austria), **U-AUT**: Upper Austria (Austria), **ESP**: Spain, **ITA-1**: North Italy (Modena), **ITA-2**: North Italy (Ravenna), **N-CRO**: North Croatia, **NTY**: North Tyrol, **S**-**POL**: South Poland, **POR**: Portugal, **W-CRO**: West Croatia.
(XLS)

Table S11 **Pairwise** ***F*** **_ST_ distances between East Tyrolean municipalities.** Pairwise comparisons between the East Tyrolean municipalities were derived from Y-STR haplotypes. *F*_ST_ values, as computed with Arlequin, are shown below and *P* values (10,040 permutations) above the diagonal.
(XLS)
