# Supplementary figures and images for: Pasture Names with Romance and Slavic Roots Facilitate Dissection of Y Chromosome Variation in an Exclusively German-Speaking Alpine Region
Source: PLoS One. 2012 Jul 27;7(7):e41885. doi: 10.1371/journal.pone.0041885 (PMC3407130; doi:10.1371/journal.pone.0041885)

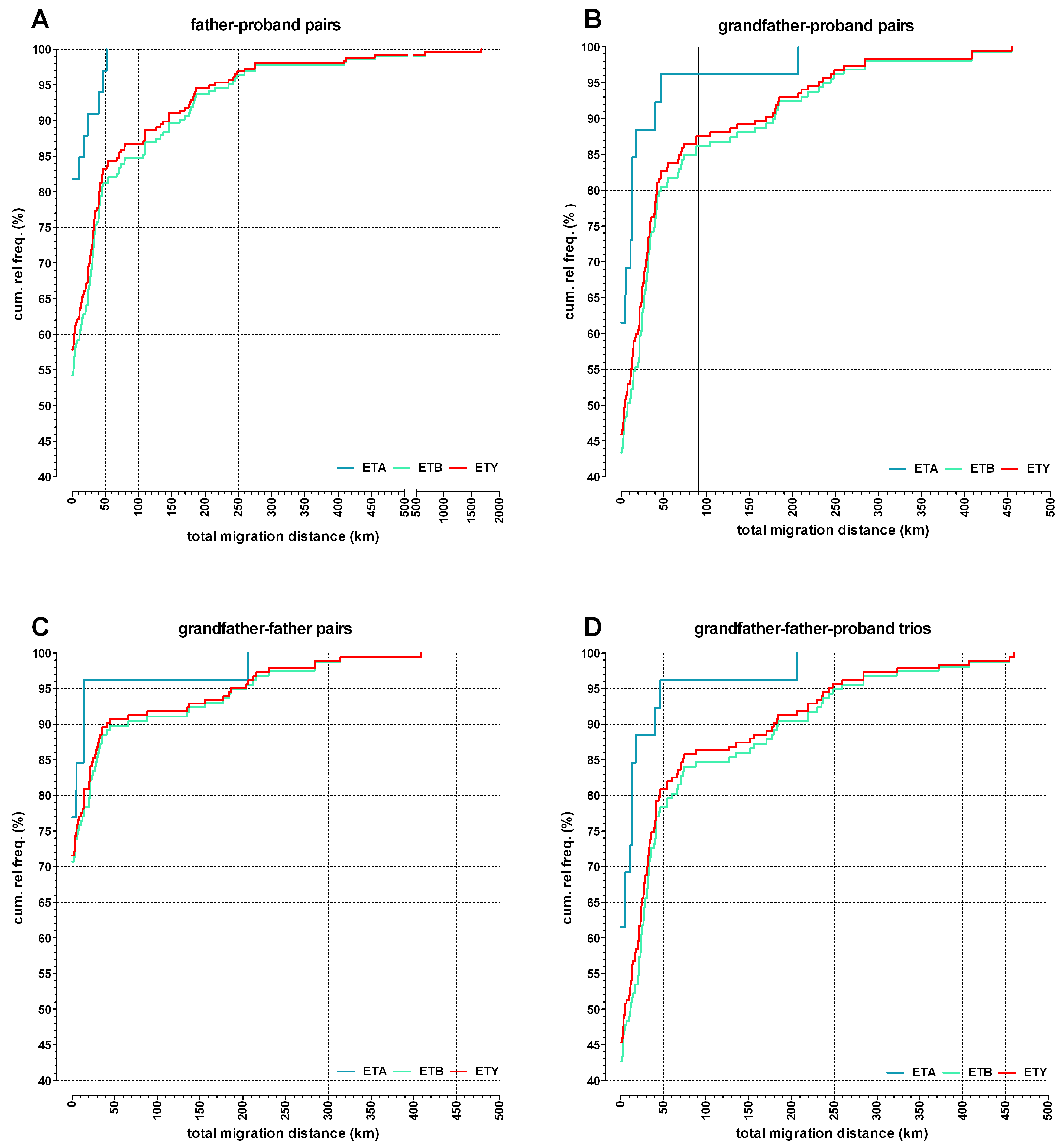

Supplement: Figure S2 — Intergenerational migration distances. Father-proband (panel A), grandfather-proband (panel B), grandfather-father (panel C), and grandfather-father-proband (panel D) migration distances were determined as present-day road distances between the probands', their fathers', and paternal grandfathers' places of birth/residence. ETA: East Tyrol region A; ETB: East Tyrol region B; ETY: East Tyrol. (TIF) [file pone.0041885.s002.tif]

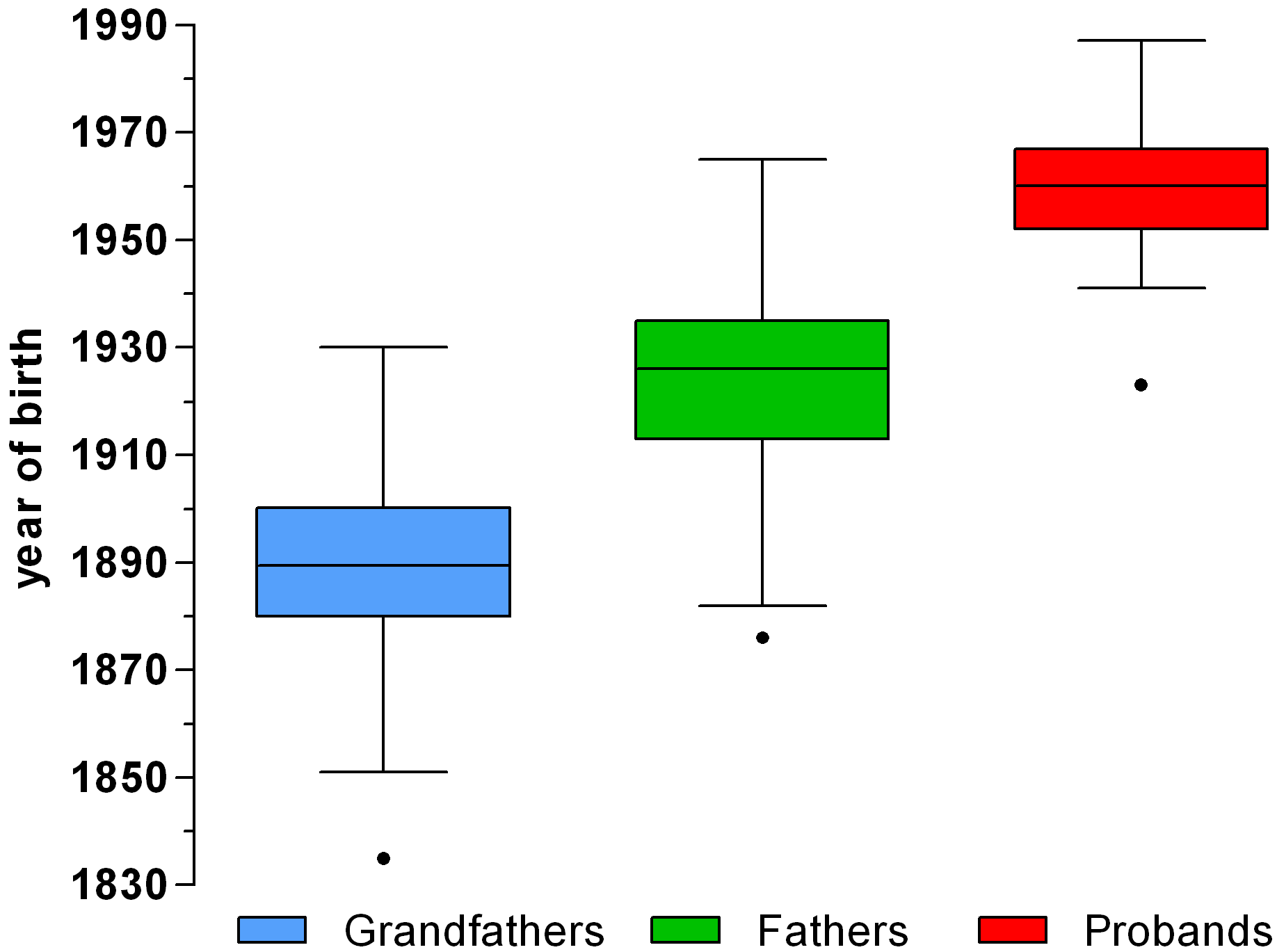

Supplement: Figure S3 — Year of birth statistics. Box-and-Whiskers plots summarizing the probands', their fathers', and paternal grandfathers' year of birth statistics. The whiskers depict the lowest (highest) datum falling within the 1.5×(Q3-Q1) interquartile range below (above) the first (third) quartile. Data points outside the 1.5 interquartile ranges are shown as dots. (TIF) [file pone.0041885.s003.tif]

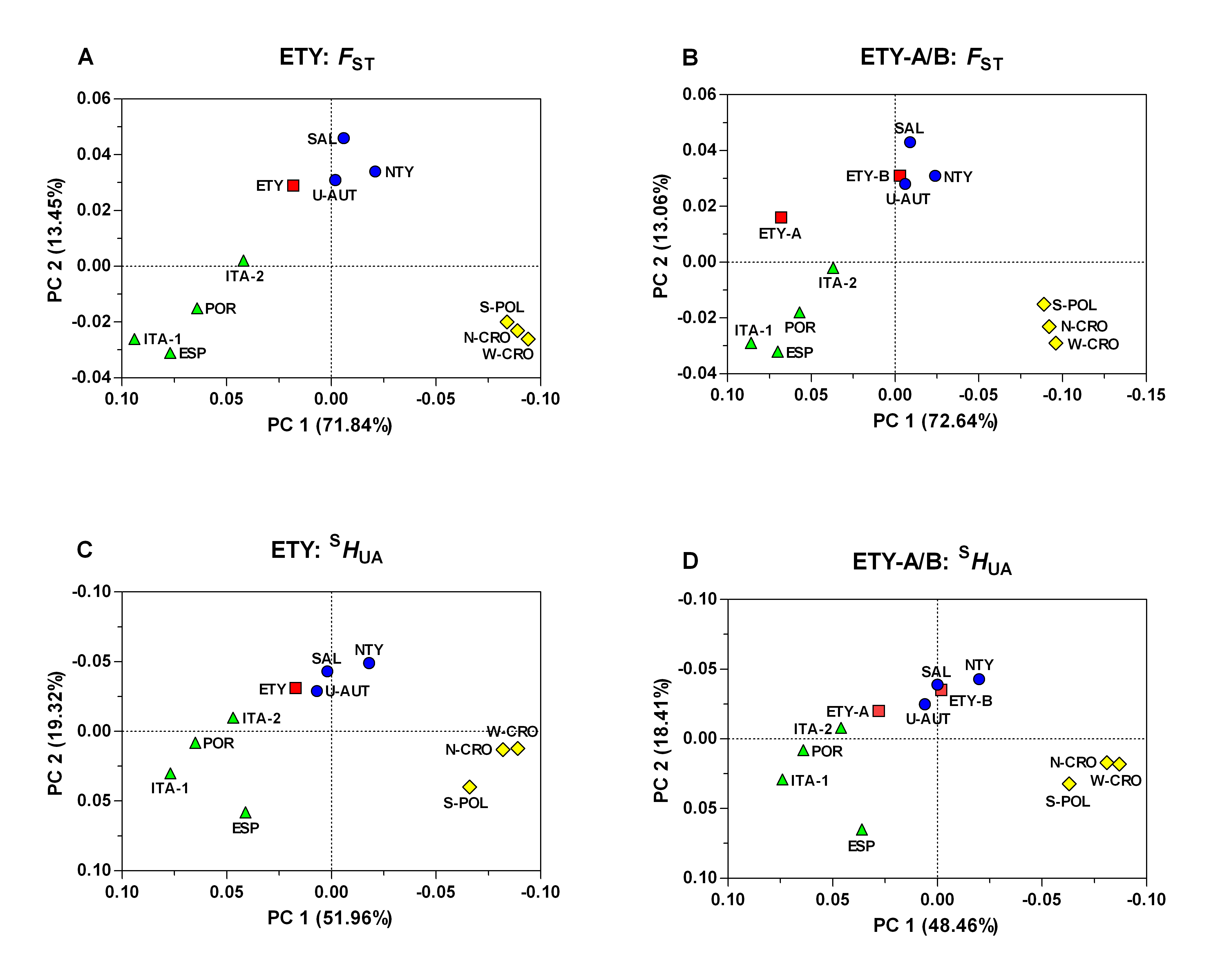

Supplement: Figure S5 — Principal coordinates analysis at the fathers-level. Plots showing the first and second principal coordinates determined by PCA using both pairwise F ST (panels A, B) and S H UA (panels C, D) distances, as obtained for the Y-STR haplotypes comprised in the combined and subdivided East Tyrolean population sample and a set of ten reference datasets. For analyses, the East Tyrolean profiles were assigned to the fathers' places of birth/residence, as reported by the probands. ETY: East Tyrol, ETY-A: East Tyrol region A, ETY-B: East Tyrol region B. Reference populations: SAL: Salzburg (Austria), U-AUT: Upper Austria (Austria), ESP: Spain, ITA-1: North Italy (Modena), ITA-2: North Italy (Ravenna), N-CRO: North Croatia, NTY: North Tyrol, S-POL: South Poland, POR: Portugal, W-CRO: West Croatia. Green triangles, blue circles and yellow diamonds indicate Romance, Germanic and Slavic language family membership of the reference populations, respectively. (TIF) [file pone.0041885.s005.tif]

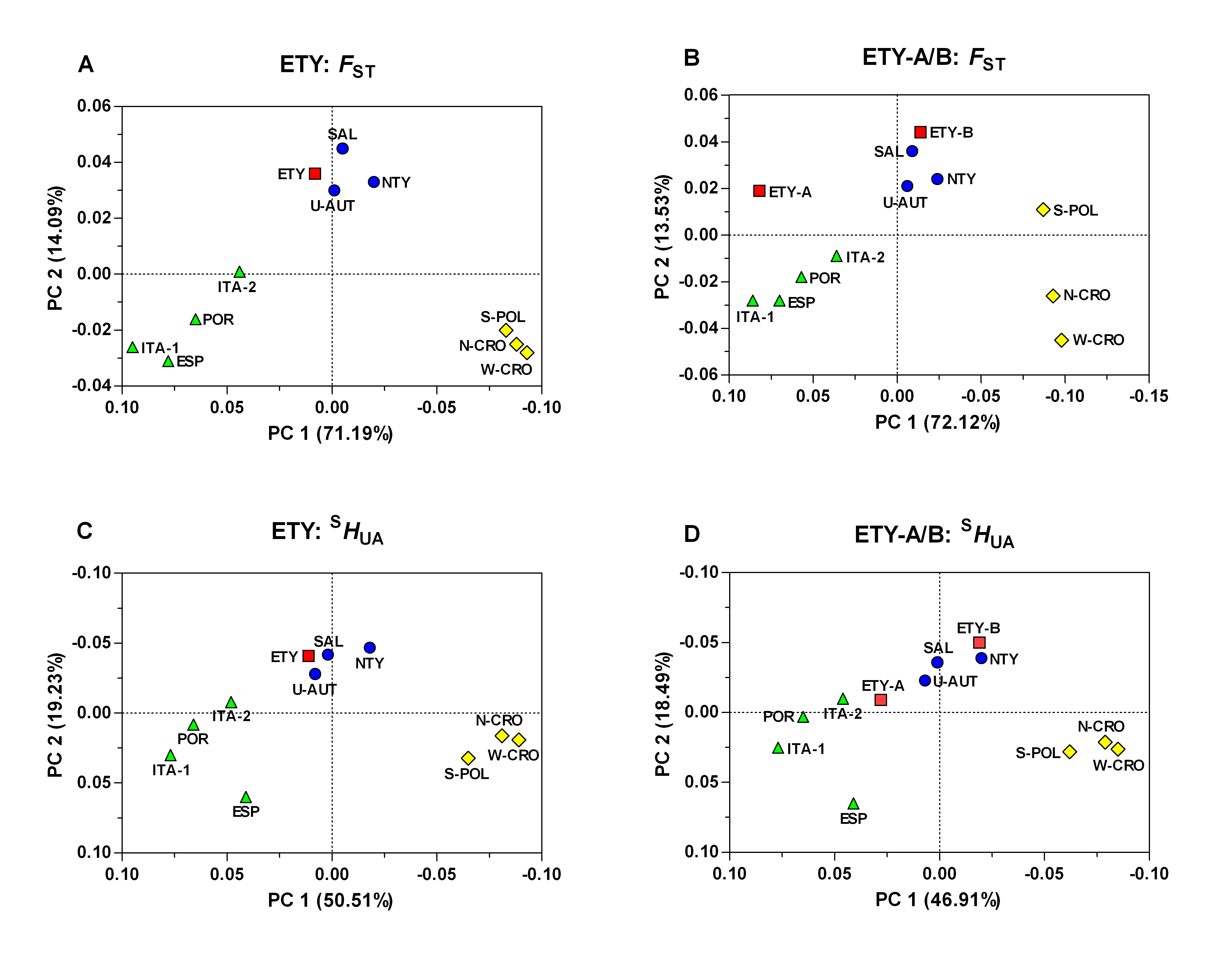

Supplement: Figure S6 — Principal coordinates analysis at the grandfathers-level. Plots showing the first and second principal coordinates determined by PCA using both pairwise F ST (panels A, B) and S H UA (panels C, D) distances, as obtained for the Y-STR haplotypes comprised in the combined and subdivided East Tyrolean population sample and a set of ten reference datasets. For analyses, the East Tyrolean profiles were assigned to the paternal grandfathers' places of birth/residence, as reported by the probands. ETY: East Tyrol, ETY-A: East Tyrol region A, ETY-B: East Tyrol region B. Reference populations: SAL: Salzburg (Austria), U-AUT: Upper Austria (Austria), ESP: Spain, ITA-1: North Italy (Modena), ITA-2: North Italy (Ravenna), N-CRO: North Croatia, NTY: North Tyrol, S-POL: South Poland, POR: Portugal, W-CRO: West Croatia. Green triangles, blue circles and yellow diamonds indicate Romance, Germanic and Slavic language family membership of the reference populations, respectively. (TIF) [file pone.0041885.s006.tif]
