# Supplementary material for: Pasture Names with Romance and Slavic Roots Facilitate Dissection of Y Chromosome Variation in an Exclusively German-Speaking Alpine Region
Source: PLoS One. 2012 Jul 27;7(7):e41885. doi: 10.1371/journal.pone.0041885 (PMC3407130; doi:10.1371/journal.pone.0041885)
Supplement: Table S2 — Amplification/sequencing primers. The sequences of the primers used for PCR amplification and sequencing were either taken from the literature [51], [65]–[69] or designed in-house. (DOC) [file pone.0041885.s008.doc]

**Table S2. Amplification/sequencing primers**.

| **Oligonucleotide** | **Sequence (5’→3’)** | **nM** | **Task** |
| --- | --- | --- | --- |
| M9 F^[65]^ | AGGACCCTGAAATACAGAACTG | 150 | SNPE |
| M9 R^[66]^ | TGCATAATGAAGTAAGCGCTACCT | 150 | SNPE |
| M17 F^[65]^ | CCTGGTCATAACACTGGAAATC | 250 | SNPE |
| M17 R^[65]^ | AGCTGACCACAAACTGATGTAGA | 250 | SNPE |
| M45 F^[65]^ | GAGAGAGGATATCAAAAATTGGCAGT | 400 | SNPE |
| M45 R^[65]^ | TGACAGTGGCACCAAAGGTC | 400 | SNPE |
| M78 F^[66]^ | TCGACATGAACACAAATTGATACACTT | 400 | SNPE |
| M78 R^[67]^ | ATAGTGTTCCTTCACCTTTCCTT | 400 | SNPE |
| M89 F | AGCTTCCTGGATTCAGCTCTCTTCCT | 300 | SNPE |
| M89 R | TGTGTGAAGTCTTGGCAGAA | 300 | SNPE |
| M96 F^[65]^ | CCACCCACTTTGTTGCTTTG | 250 | SNPE |
| M96 R^[65]^ | TGCCCTCTCACAGAGCACTT | 250 | SNPE |
| M170 F^[65]^ | CAGCTCTTATTAAGTTATGTTTTCATATTCTGTG | 500 | SNPE |
| M170 R^[65]^ | GTCCTCATTTTACAGTGAGACACAAC | 500 | SNPE |
| M173 F^[65]^ | TTTTCTTACAATTCAAGGGCATTTAG | 300 | SNPE |
| M173 R^[65]^ | CTGAAAACAAAACACTGGCTTATCA | 300 | SNPE |
| M201 F^[65]^ | TCAGATCTAATAATCCAGTATCAACTGAG | 400 | SNPE |
| M201 R^[68]^ | GTTCAAATCCCATATCCAGCA | 400 | SNPE |
| M223 F | TTTCTGGTGTCTAGGAAAGTCGTG | 300 | SNPE |
| M223 R^[66]^ | CAGCAAGAGTAAGCAAGAGGCACT | 300 | SNPE |
| M253 F | GCTGATCTGTTTCTTTTTGGTGTT | 400 | SNPE |
| M253 R | TCCAACTCTATGCAGTTTATGACCA | 400 | SNPE |
| M269 F | CATGCCTAGCCTCATTCCTC | 300 | SNPE |
| M269 R | GCCCGGCCACTATACTTCTT | 300 | SNPE |
| M304 F^[68]^ | CAAAGAAAAGCAGGAGAGTTTGTAA | 300 | SNPE |
| M304 R^[68]^ | AAACGTCTTATACCAAAATATCACCAG | 300 | SNPE |
| M343 F | CTGATTCGCACAAGGCTCAG | 200 | SNPE |
| M343 R | TCACTCAACATGGCGATTCC | 200 | SNPE |
| P15 F^[69]^ | AGAGAGTTTTCTAACAGGGCG | 200 | SNPE |
| P15 R | CCAGCCCACTTGTGGACTTTAT | 200 | SNPE |
| P37 F | CATAGTGATAGGGTGGGATTGGTT | 500 | SNPE |
| P37 Y R | TGAGCCAGACTCTCAGGTTAAGT | 500 | SNPE |
| SRY_10831_ F^[65]^ | CCACATAGGTGAACCTTGAAAATG | 400 | SNPE |
| SRY_10831_ R^[65]^ | TCATCCAGTCCTTAGCAACCATTA | 400 | SNPE |
| U106 F | GACATGCTCTGGTGCATAGGGATT | 150 | SNPE |
| U106 R | TGGCAGATGCAAGCTACCTA | 150 | SNPE |
| U152 F | GCCTCTTTTTGGCTTCCATA | 200 | SNPE |
| U152 R | GAGAAACATTCCACGCTTGAG | 200 | SNPE |
| L11 F* | CCCAGGCATCAACCTCTGAAGGC | 300 | CTS |
| L11 R | ACTGGCTTCTTCAAAGCACCCACA | 300 | CTS |
| L23 F^[51]^ | CCTGTAGTCCCAGCTACTCAGG | 300 | CTS |
| L23 R*^[51]^ | TATCCAATCTGCCATCAGTG | 300 | CTS |
| M20 F* | TGGCCCTTTGTGTCTGTGAGTTTC | 300 | CTS |
| M20 R* | CCCACACCACCTGCAAATACCAAA | 300 | CTS |
| M70 F* | TCACGTCTTCCTCAATGCTGCTCA | 300 | CTS |
| M70 R* | ACAGGCTACCCAGGAGTACAGTTT | 300 | CTS |
| M242 F* | TCCACTGACGACGTATTAACGCCT | 300 | CTS |
| M242 R* | TACCTAGAACAACTCTGAAGCGGTGG | 300 | CTS |
| M412 F^[51]^ | AGACACTAGCAGTCTTGTCCTCAG | 300 | CTS |
| M412 R*^[51]^ | CAGGTATGGAAGTGCTCAAATCG | 300 | CTS |
| M529 F^[51]^ | TCTTATCATTGTCACAGGGCTG | 300 | CTS |
| M529 R*^[51]^ | TGCCTTAGCTGTCTGTGTCC | 300 | CTS |
| S116 F*^[51]^ | TTTCTCAACCCACTGTCTGC | 300 | CTS |
| S116 R^[51]^ | TCATGCCATTGTACTCCAGC | 300 | CTS |

**F**(orward) and **R**(everse) primer designations relate to the Y-chromosome + strand orientation

**SNPE**: multiplex PCR for single nucleotide primer extension

**CTS**: singleplex PCR for chain termination sequencing
*: primer also used in sequencing reactions [160 nM]

The sequences of the primers for PCR amplification and sequencing were either taken from the literature or designed in-house.
