# Supplementary material for: Pasture Names with Romance and Slavic Roots Facilitate Dissection of Y Chromosome Variation in an Exclusively German-Speaking Alpine Region
Source: PLoS One. 2012 Jul 27;7(7):e41885. doi: 10.1371/journal.pone.0041885 (PMC3407130; doi:10.1371/journal.pone.0041885)
Supplement: Table S3 — Oligonucleotides used for 19-plex single nucleotide primer extension. The primer sequences were either taken from the literature [65]–[68], [70] or designed in-house. (DOC) [file pone.0041885.s009.doc]

**Table S3. Oligonucleotides used for 19-plex single nucleotide primer extension.**

| **Oligonucleotide** | **Sequence (5'→3')** | **nt** |
| --- | --- | --- |
| P37_SNPE_ F | CT**TAGGGTGGGATTGGTTCA** | 20 |
| M89_SNPE_ R | T**CAACTCAGGCAAAGTGAGAGAT** | 23 |
| U106_SNPE_ F | (GACT)_2_**GCAAATCCCAAAGCTCCA** | 26 |
| M45_SNPE_ R^[65,68]^ | (GACT)_3_T**CTCAGAAGGAGCTTTTTGC** | 32 |
| SRY10831_SNPE_ R | (GACT)_2_**TCTTGTATCTGACTTTTTCACACAGT** | 34 |
| P15_SNPE_ F | ACT(GACT)_2_GAC**ATGCTTGAGGTTCTGAATCTTA** | 36 |
| M269_SNPE_ F^[66]^ | ACT(GACT)_3_GA**GGAATGATCAGGGTTTGGTTAAT** | 40 |
| M17_SNPE_.R^[65,67,68]^ | (GACT)_5_GA**CCAAAATTCACTTAAAAAAACCC** | 45 |
| M223_SNPE_ R | (GACT)_5_GA**CTGCACATTGATAAATTTACTTACAGT** | 49 |
| M253_SNPE_ F | (GACT)_7_**GTATTGTTGATAGATAGCAAGTTGA** | 53 |
| U152_SNPE_ F | (GACT)_7_GAC**TCTATACATTACTTTGAGAAGTATGG** | 57 |
| M78_SNPE_ R^[66]^ | (GACT)_9_G**TTTTGAAATATTTGGAAGGGC** | 58 |
| M343_SNPE_ R | (GACT)_11_T**CCACATATCTCCAGGTGT** | 63 |
| M304_SNPE_ F^[68]^ | (GACT)_10_**TGTTCAATTTGAAAGTAACTTGTGA** | 65 |
| M201_SNPE_ F^[68]^ | (GACT)_10_**GATCTAATAATCCAGTATCAACTGAGG** | 67 |
| M173_SNPE_ F | (GACT)_10_GAC**TCTTACAATTCAAGGGCATTTAGAAC** | 69 |
| M170_SNPE_ F^[68]^ | (GACT)_11_**CTATTTTATTTACTTAAAAATCATTGTTC** | 73 |
| M9_SNPE_ F^[66,70]^ | (GACT)_14_G**ACGGCCTAAGATGGTTGAAT** | 77 |
| M96_SNPE_ F | (GACT)_14_**CTTGGAAAACAGGTCTCTCATAATA** | 81 |

**F**(orward) and **R**(everse) primer designations relate to the Y-chromosome + strand orientation

Target-specific sequences are indicated by boldface letters, **nt**: nucleotides

The primer sequences were either taken from the literature or designed in-house.
