# Supplementary material for: Pasture Names with Romance and Slavic Roots Facilitate Dissection of Y Chromosome Variation in an Exclusively German-Speaking Alpine Region
Source: PLoS One. 2012 Jul 27;7(7):e41885. doi: 10.1371/journal.pone.0041885 (PMC3407130; doi:10.1371/journal.pone.0041885)
Supplement: Table S4 — Amplicon sequences. Overview of the sequences of all PCR products generated for genotyping the haplogroup diagnostic Y-SNPs [11], [51], [59], [60], [71]–[80]. All sequences are shown in Y chromosome+strand orientation. PCR primer binding regions are depicted by upper case, bold face letters, those for single base extension primers are underlined. SNP positions are shown in parentheses. (DOC) [file pone.0041885.s010.doc]

**Table S4. Amplicon sequences.**

***L11/S127***^[51]^

**CCCAGGCATCAACCTCTGAAGGC**ttgtactcttgtaatgtggtgtcttacctgtgggcatttgtaagagaatgaatagaagatgaagtactctacttagacatgtgtgatgtctttctccacctaggtttctgtgagaaaactaa**(T>C)**gaagaacttttggttctgtctgttctaagagacaatccttgctctctccaggaatttcactaaaacagga**TGTGGGTGCTTTGAAGAAGCCAGT**

***L23/S141***^[51]^

**CCTGTAGTCCCAGCTACTCAGG**aggctgaggcaggagaatggcatgagcccaggaggcagagcttgcagggagccaaaattgtgccactgcactccagcctgggcgacagagcgagactct**(G>A)**tctcaaaggagtttaaaacagaaatatcctttgtccccacaatcttattactagccatctacccaaatgaatataatttgttttgccatataagtcacatttgcacatatatttatcacagtactgttcataatatcaaacacatgaaatcaatgtagatgcc**CACTGATGGCAGATTGGATA**

**M9**^[71]^

**AGGACCCTGAAATACAGAACTG**caaagaaacggcctaagatggttgaat**(C>G)**ctctttatttttctttaatttagacatgttcaaacgttcaatgtcttacatacttagttatgtaagta**AGGTAGCGCTTACTTCATTATGCA**

**M17**^[71]^, M18^[71]^ (rs3909), *M19*^[71]^ (rs3910)

**CCTGGTCATAACACTGGAAATC**agattctgtctactcaccagagtttgtggttgctggttgtt(a>aaa)c**(GGGG>GGG)**tttttttaagtgaattttggggtttgttaagtggccaaactatttttgtgaagactgttgta(*t>a*)gtgggtttcagatgtc**TCTACATCAGTTTGTGGTCAGCT**

***M20***^[71]^

**TGGCCCTTTGTGTCTGTGAGTTTC**acatttgtaggttcaaccaactgtggattgaaaat**(A>G)**tttgaaaaattaaaaatagatggttgcatttgcactgaacatgtagacttttttttcttgtaatttctcttaaaccatacagcataacaactctttacatagcatgtacattgtattaggtattctgagtactctaaagtatacgggaggatgtgtgtaggttatgtgcaaatactataacattatatgtaagggatttgaaaattctgggat**TTTGGTATTTGCAGGTGGTGTGGG**

**M45**^[72]^

**GAGAGAGGATATCAAAAATTGGCAGT**gaaaaattatagata**(G>A)**gcaaaaagctccttctgaggtccaggccaggagatagtaggatttaagaaacaaacaaacaaaaacaaccacaaat**GACCTTTGGTGCCACTGTCA**

***M70***^[73]^

**TCACGTCTTCCTCAATGCTGCTCA**ccagcctccagaattccttctctacaagttctgtaggcaccatctgtgaaaacacatgtaaaaggttatcatagcccactatactttggactcatgtctccatgaga**(A>C)**ctaagactaccacaacagaatccctatagtccagccctcagatcacatacatgtacaggcatgttgaagtagtcggacttgaaggaatcagccatttcaccaaaactctgc**AAACTGTACTCCTGGGTAGCCTGT**

**M78**^[74]^

**TCGACATGAACACAAATTGATACACTT**aacaaagatacttctttc**(C>T)**gcccttccaaatatttcaaaataagctggtcatagtacttgcttttcataaaaagatggtaagcttccaatatttagattt**AAGGAAAGGTGAAGGAACACTAT**

**M89**^[72]^

**AGCTTCCTGGATTCAGCTCTCTTCCT**aaggttatgtacaaaaatct**(C>T)**atctctcactttgcctgagttgcagctacctttgctggtgatcctggacccaaagtgtgccagcctctcctgatactctgtgtgtacctgagcagctattc**TGCCAAGACTTCACACA**

**M96**^[74]^

**CCACCCACTTTGTTGCTTTG**taaaggaccatatattttgccataggtttttaatattatacctgagtgttttatc**(C>G)**tattatgagagacctgttttccaagttacacatcacagctcacttta**AAGTGCTCTGTGAGAGGGCA**

**M170**^[74]^

**CAGCTCTTATTAAGTTATGTTTTCATATTCTGTG**cattatacaaattactattttatttacttaaaaatcattgttc**(A>C)**tttttttcagtgtgg**GTTGTGTCTCACTGTAAAATGAGGAC**

**M173**^[74]^

**TTTTCTTACAATTCAAGGGCATTTAG**aac**(A>C)**ctttgtcatctgttaatattcagaaa**TGATAAGCCAGTGTTTTGTTTTCAG**

**M201**^[73]^

**TCAGATCTAATAATCCAGTATCAACTGAG**g**(G>T)**ttttcgtaataggtacttagtgttggatgaagctgatagga**TGCTGGATATGGGATTTGAAC**

**M223**^[73]^

**TTTCTGGTGTCTAGGAAAGTCGTG**gaggcaagtatgccgctataaaaattagactctgtgttactagctgaagatgatgcaatttatttac**(G>A)**actgtaagtaaatttatcaatgtgcagactccagcggctc**AGTGCCTCTTGCTTACTCTTGCTG**

***M242***^[75]^

**TCCACTGACGACGTATTAACGCCT**gcagtttactgagacttgacgtgttaaatagaccacagaaatagaaaattaagagcctgggcaatacgcatttatgttttctatgtaattggcatccctttaactcttgataaaccgtgctgtctagttcactagaattaagtagtaaattcagatgrcaagatttttaagtacagtagtatcttaattgatgattcatgtaatgtgatagtatcttgaacttatatatgtaagctttctacggcatagaaagtttgtgcaaaaaggtgaccaaggtgct**(C>T)**ttggcattggtcttaacgtgttttttgaaaaaaatctattttaacgtacatggttttttcccccacccccg**CCACCGCTTCAGAGTTGTTCTAGGTA**

**M253**^[76]^

**GCTGATCTGTTTCTTTTTGGTGTT**actagacaaagctagattttaaaagatgaattaagatgctcagctaactagtcctgtttatagtattgttgatagatagcaagttga**(C>T)**ttctccaggttcttcattgaatgagtccttgtttactatgatgcttgctacatacagttgctacatactactatgtatgagtagtttt**TGGTCATAAACTGCATAGAGTTGGA**

**M269**^[77]^

**CATGCCTAGCCTCATTCCTC**taaaatataatttaaagtggattctgttacatggtatcacaatagaaggggaatgatcagggtttggttaat**(T>C)**ctggtaaattgaaaacaattttttttttatcatatgtgcctcagaaggcacacaa**AAGAAGTATAGTGGCCGGGC**

**M304**^[76]^

**CAAAGAAAAGCAGGAGAGTTTGTAA**caaacagtatgtgggatttttttagatgtgttcaatttgaaagtaacttgtga**(A>C)**acaa**CTGGTGATATTTTGGTATAAGACGTTT**

**M343**^[76]^

**CTGATTCGCACAAGGCTCAG**ggtattggtttgaccaggcatgtctttcacgtagcccgagagaaaactggccaccctagccttttaaatatgcaaatgcagagtgccctcgtgttcca**(C>A)**acacctggagatatgtgggggtggctatgctgccaggcacgtgttggggaaagagcaagaggacaaaggtg**GGAATCGCCATGTTGAGTGA**

***M412/S167***^[51]^

**AGACACTAGCAGTCTTGTCCTCAG**ggccccttgaatttcctcaaattctgttcccagctgaacaggtgcttcattgtgtgagggggcactcctccgtcatcttgggatttcatcctgggatagagagtgtgagaagaaataaggtgagatatggacgggggtacaatctgatgaggc**(G>A)**tggatagggtccacttcacctgtaaaatacatgaagatagatgacatagaaggtgcttccaaatccattcccacatttacttaattgaacaagccatcccacatcatggctcagtgttcaggcaggaatactccaatgtccaattatcatttggagtgcaaacttgtggcatcctatcaaactcc**CGATTTGAGCACTTCCATACCTG**

***M529/S145***^[51]^

**TCTTATCATTGTCACAGGGCTG**attctctaaaattgaccatgtagtcagacattaaaccctcctcagcaacagtaaaacagatgaaattataacaaccgctctctcagaca**(C>G)**cagcgcaataaaattgggaatcaagactaataaatttactgaaaaccatacaattacatggaaattgaataacctgcttctgaatgcttccaggtaaataatgacattaaggcataaatcaagaacctccttgaaacaaatgagaacaaagatacaacataccagaatctctt**GGACACAGACAGCTAAGGCA**

**P15**^[11]^

**CAGAGAGTTTTCTAACAGGGCG**taacaaataggttaaattatgacttacaggcacactcaacagacagtgaagatgacagtaaagattatcatttcctcacatgaatagagccaatgcttgaggttctgaatctta**(C>T)**gcctgaaggcagatgaaagttgcaaaagtgattcccatatttag**ATAAAGTCCACAAGTGGGCTGG**

**P37** (**.1**: T>C; **.2**: T>C)^[59]^

**CATAGTGATAGGGTGGGATTGGTT**ca**(Y)**agtgtaaaccaaaaataaatttaaaggccccccacaaccatctgaatggattcccgcctttgccagggcaccctaaa**ACTTAACCTGAGAGTCTGGCTCA**

***S116***^[51]^

**TTTCTCAACCCACTGTCTGC**ctttaagtttataccaaacacaagtgatggtggctaattcttttacttgggcaactttggaatacgtattttcttatttttatttaggtggtgtcaatttattttcaatgttatgattaaggtcctgctaatgtatctgctgcactg**(C>A)**ctttcactttagccccaactccacccccaccaaggcatcttgtgtcttctgctcatggttgaaaaatcagcactgaggtgataaatgagcactggatctgaagtcagctttgttttcactgaatttctaagtgcctcactcccttgccctg**GCTGGAGTACAATGGCATGA**

**SRY_10831_** (**.1**: T>C; **.2**: C>T)^[78]^

**CCACATAGGTGAACCTTGAAAATG**tta**(Y)**actgtgtgaaaaagtcagatacaagaggccagatactgaatgatcccatgtatatgaaatatccaggaaaggcaaatgaatagagactaaaaccagat**TAATGGTTGCTAAGGACTGGATGA**

**U106/S21**^[60,79^**,**^80]^

**GACATGCTCTGGTGCATAGGGATT**cctgaatagcaaatcccaaagctcca**(C>T)**gggttcaattgcttcttctctgaatacctgccttccccgaccaaacacacaggaagacacatacagctgctaggttttatttccctccaggtgtcatttcaggtccatgacatctcctc**TAGGTAGCTTGCATCTGCCA**

**U152/S28**^[60]^

**GCCTCTTTTTGGCTTCCATA**tgattttaaaatatgttttccaattatctgaagaatgtcaatggtagtttaatgggagtagcattgaatctatacattactttgagaagtatgg**(C>T)**tattttcacaatactcatttttcttatc**CTCAAGCGTGGAATGTTTCTC**

Overview of the sequences of all PCR products generated for genotyping the haplogroup diagnostic Y-SNPs. All sequences are shown in Y chromosome + strand orientation. PCR primer binding regions are depicted by upper case, bold face letters, those for single base extension primers are underlined. SNP positions are shown in parentheses. Markers that were genotyped by Sanger sequencing are indicated by italicised typeface.
